# Identification of sixteen novel candidate genes for late onset Parkinson’s disease

**DOI:** 10.1186/s13024-021-00455-2

**Published:** 2021-06-21

**Authors:** Alessandro Gialluisi, Mafalda Giovanna Reccia, Nicola Modugno, Teresa Nutile, Alessia Lombardi, Luca Giovanni Di Giovannantonio, Sara Pietracupa, Daniela Ruggiero, Simona Scala, Stefano Gambardella, International Parkinson’s Disease Genomics Consortium (IPDGC), Alastair J. Noyce, Rauan Kaiyrzhanov, Ben Middlehurst, Demis A. Kia, Manuela Tan, Henry Houlden, Huw R. Morris, Helene Plun-Favreau, Peter Holmans, John Hardy, Daniah Trabzuni, John Quinn, Vivien Bubb, Kin Y. Mok, Kerri J. Kinghorn, Kimberley Billingsley, Nicholas W. Wood, Patrick Lewis, Sebastian Schreglmann, Ruth Lovering, Lea R’Bibo, Claudia Manzoni, Mie Rizig, Mina Ryten, Sebastian Guelfi, Valentina Escott-Price, Viorica Chelban, Thomas Foltynie, Nigel Williams, Karen E. Morrison, Carl Clarke, Alexis Brice, Fabrice Danjou, Suzanne Lesage, Jean-Christophe Corvol, Maria Martinez, Claudia Schulte, Kathrin Brockmann, Javier Simón-Sánchez, Peter Heutink, Patrizia Rizzu, Manu Sharma, Thomas Gasser, Mark R. Cookson, Sara Bandres-Ciga, Cornelis Blauwendraat, David W. Craig, Derek Narendra, Faraz Faghri, J. Raphael Gibbs, Dena G. Hernandez, Kendall Van Keuren-Jensen, Joshua M. Shulman, Hirotaka Iwaki, Hampton L. Leonard, Mike A. Nalls, Laurie Robak, Jose Bras, Rita Guerreiro, Steven Lubbe, Steven Finkbeiner, Niccolo E. Mencacci, Codrin Lungu, Andrew B. Singleton, Sonja W. Scholz, Xylena Reed, Roy N. Alcalay, Ziv Gan-Or, Guy A. Rouleau, Lynne Krohn, Lynne Krohn, Jacobus J. van Hilten, Johan Marinus, Astrid D. Adarmes-Gómez, Miquel Aguilar, Ignacio Alvarez, Victoria Alvarez, Francisco Javier Barrero, Jesús Alberto Bergareche Yarza, Inmaculada Bernal-Bernal, Marta Blazquez, Marta Bonilla-Toribio, Juan A. Botía, María Teresa Boungiorno, Dolores Buiza-Rueda, Fátima Carrillo, Mario Carrión-Claro, Debora Cerdan, Jordi Clarimón, Yaroslau Compta, Monica Diez-Fairen, Oriol Dols-Icardo, Jacinto Duarte, Raquel Duran, Francisco Escamilla-Sevilla, Mario Ezquerra, Cici Feliz, Manel Fernández, Rubén Fernández-Santiago, Ciara Garcia, Pedro García-Ruiz, Pilar Gómez-Garre, Maria Jose Gomez Heredia, Isabel Gonzalez-Aramburu, Ana Gorostidi Pagola, Janet Hoenicka, Jon Infante, Silvia Jesús, Adriano Jimenez-Escrig, Jaime Kulisevsky, Miguel A. Labrador-Espinosa, Jose Luis Lopez-Sendon, Adolfo López de Munain Arregui, Daniel Macias, Irene Martínez Torres, Juan Marín, Maria Jose Marti, Juan Carlos Martínez-Castrillo, Carlota Méndez-del-Barrio, Manuel Menéndez González, Marina Mata, Adolfo Mínguez, Pablo Mir, Elisabet Mondragon Rezola, Esteban Muñoz, Javier Pagonabarraga, Pau Pastor, Francisco Perez Errazquin, Teresa Periñán-Tocino, Javier Ruiz-Martínez, Clara Ruz, Antonio Sanchez Rodriguez, María Sierra, Esther Suarez-Sanmartin, Cesar Tabernero, Juan Pablo Tartari, Cristina Tejera-Parrado, Eduard Tolosa, Francesc Valldeoriola, Laura Vargas-González, Lydia Vela, Francisco Vives, Alexander Zimprich, Lasse Pihlstrom, Mathias Toft, Sulev Koks, Pille Taba, Sharon Hassin-Baer, Kari Majamaa, Ari Siitonen, Njideka U. Okubadejo, Oluwadamilola O. Ojo, Rauan Kaiyrzhanov, Chingiz Shashkin, Nazira Zharkynbekova, Vadim Akhmetzhanov, Akbota Aitkulova, Elena Zholdybayeva, Zharkyn Zharmukhanov, Gulnaz Kaishybayeva, Altynay Karimova, Dinara Sadykova, Licia Iacoviello, Fernando Gianfrancesco, Dario Acampora, Maurizio D’Esposito, Antonio Simeone, Marina Ciullo, Teresa Esposito

**Affiliations:** 1 IRCCS Istituto Neurologico Mediterraneo Neuromed, Pozzilli, Isernia, Italy; 2 National Research Council, Institute of Genetics and Biophysics “Adriano Buzzati-Traverso”, Naples, Italy; 3 Department of Biomolecular Science, University of Urbino Carlo Bò, Urbino, Italy; 4 Research Center in Epidemiology and Preventive Medicine (EPIMED), Department of Medicine and Surgery, University of Insubria, Varese, Italy

**Keywords:** Late onset Parkinson’s disease, Whole exome sequencing, Novel candidate genes for Parkinson’s disease, Rare variant burden analysis

## Abstract

**Background:**

Parkinson’s disease (PD) is a neurodegenerative movement disorder affecting 1–5% of the general population for which neither effective cure nor early diagnostic tools are available that could tackle the pathology in the early phase. Here we report a multi-stage procedure to identify candidate genes likely involved in the etiopathogenesis of PD.

**Methods:**

The study includes a discovery stage based on the analysis of whole exome data from 26 dominant late onset PD families, a validation analysis performed on 1542 independent PD patients and 706 controls from different cohorts and the assessment of polygenic variants load in the Italian cohort (394 unrelated patients and 203 controls).

**Results:**

Family-based approach identified 28 disrupting variants in 26 candidate genes for PD including *PARK2, PINK1, DJ-1(PARK7), LRRK2, HTRA2, FBXO7, EIF4G1, DNAJC6, DNAJC13, SNCAIP, AIMP2*, *CHMP1A, GIPC1, HMOX2, HSPA8*, *IMMT*, *KIF21B, KIF24, MAN2C1, RHOT2, SLC25A39, SPTBN1, TMEM175, TOMM22, TVP23A* and *ZSCAN21*. Sixteen of them have not been associated to PD before, were expressed in mesencephalon and were involved in pathways potentially deregulated in PD. Mutation analysis in independent cohorts disclosed a significant excess of highly deleterious variants in cases (*p* = 0.0001), supporting their role in PD.

Moreover, we demonstrated that the co-inheritance of multiple rare variants (≥ 2) in the 26 genes may predict PD occurrence in about 20% of patients, both familial and sporadic cases, with high specificity (> 93%; *p* = 4.4 × 10^− 5^). Moreover, our data highlight the fact that the genetic landmarks of late onset PD does not systematically differ between sporadic and familial forms, especially in the case of small nuclear families and underline the importance of rare variants in the genetics of sporadic PD.

Furthermore, patients carrying multiple rare variants showed higher risk of manifesting dyskinesia induced by levodopa treatment.

**Conclusions:**

Besides confirming the extreme genetic heterogeneity of PD, these data provide novel insights into the genetic of the disease and may be relevant for its prediction, diagnosis and treatment.

**Supplementary Information:**

The online version contains supplementary material available at 10.1186/s13024-021-00455-2.

## Background

Parkinson’s disease (PD) is a neurodegenerative movement disorder characterized by the loss of mesodiencephalic dopaminergic (mdDA) neurons of the substantia nigra pars compacta (SNpc), in association with the presence of Lewy bodies in some surviving neurons [1, 2]. mdDA neurons play a crucial role in the control of motor, sensory-motor and motivated behavior [3]. Their degeneration causes the characteristic symptoms of PD, which include resting tremor, bradykinesia, rigidity, postural instability and a variety of other motor and non–motor symptoms [2]. It has been demonstrated that disease’s symptoms are evident when patients have already lost 50–60% of their DA neurons, suggesting the need for early diagnosis of the disease [2]. Most PD cases are sporadic, with unknown etiology. Approximately 5–10% of PD cases appear to have monogenic forms of inheritance including autosomal dominant (*SNCA*, *LRRK2*, and *VPS35*) and recessive *(PARK2*, *PINK1*, *DJ1*) forms [4–6]. However, many additional loci associated to PD have recently been identified, even though for most of them the molecular function is still unknown [5]. Moreover, there is a high genetic heterogeneity at the basis of PD, with many different rare mutations usually detected only in a single family or in small populations [5]. Despite extensive ongoing studies, the pathogenic mechanisms underpinning PD remain largely elusive [6]. Moreover, although the Next Generation Sequencing (NGS) methods are widespread also in the diagnostic field, their application to PD is still limited to a small number of patients with a clear family history and to a small number of causative mutations, due to the high genetic heterogeneity associated with the disease and to the difficulty in interpreting test results.

In this study, we performed a multi-stage procedure aimed at the identification of potential PD-causative variants and then at demonstrating that the co-inheritance of multiple rare variants in Mendelian genes may increase the risk of PD in a non-Mendelian fashion, and that this may influence the occurrence of the disease and the manifestation of clinical signs.

## Patients and methods

### Study participants

#### PIB cohort

**Fig. 1:**
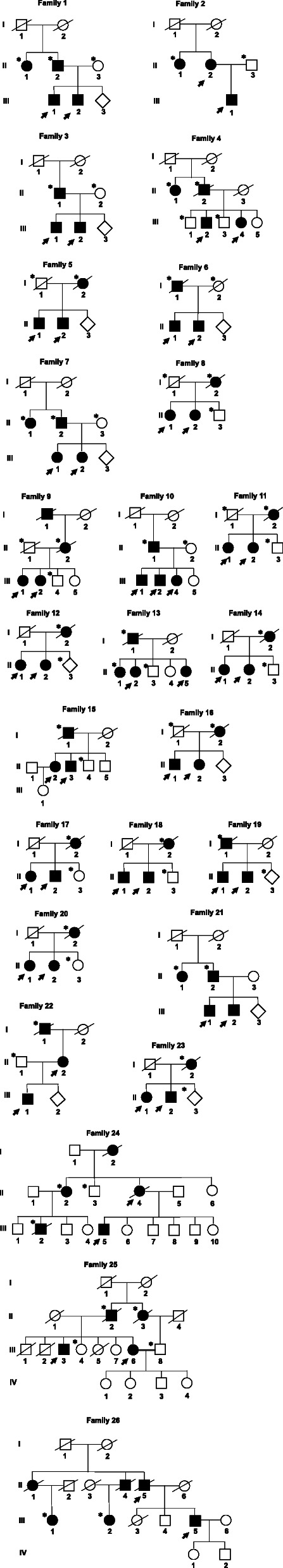
Graphical representation of the 26 families used as discovery cohort. Pedigrees of the 26 Italian families with supposedly dominant PD forms. Affected individuals are indicated with dark symbols. Patients who underwent WES analysis are shown with dark arrows. Additional family members for which DNA was available are indicated with an asterisk. Only affected subjects were used for segregation analysis. For each family, the rare deleterious variants reported in Table 1 were carried by the affected family members

Twenty-three PD families with supposedly dominant transmission (Fig. 1, families numbered 1 through 23) were recruited at the “Parkinson Institute Biobank,” member of the Telethon Network of Genetic Biobank (biobanknetwork.telethon.it/) (hereafter-called PIB cohort). Forty-seven PD patients (siblings or parent-offspring pairs) from these families underwent whole-exome sequencing (WES) analysis to identify novel PD candidate genes. Forty PD patients (of which only a small amount of DNA was available), from the same 23 families, were used for segregation analysis to confirm the variants identified through WES approach. All PD subjects (age at onset 61.7; Standard Deviation (SD) ± 8.57) were evaluated by neurologists of the Parkinson Study Group, according to published diagnostic criteria (Table S1) [7]. Written informed consent was obtained from all participants.

#### MNI cohort

Three PD families with supposedly dominant transmission were recruited at the IRCCS Mediterranean Neurological Institute (MNI) in Pozzilli (hereafter called MNI cohort) (Fig. 1; families numbered 24 through 26). Two PD cases for each family underwent WES analysis to identify novel PD candidate genes. Segregation analysis to confirm WES results was performed on PD cases from the same families (Fig. 1).

Three hundred ninety-four independent and unrelated PD patients (243 males; 172 familiar and 222 sporadic cases), were used as validation cohort, as well as to assess the polygenic inheritance of rare variants as risk factor for PD occurrence and for endophenotypes manifestation (Table S1) [8]. The project was approved by the ethical committees of IRCCS Neuromed and written informed consent was signed by all the participants. All subjects were of European ancestry and were evaluated by qualified neurologist of the Parkinson Centre from June 2015 to December 2017, with a thorough protocol comprising neurological examination and evaluation of non-motor domains. Information about family history, demographic characteristics, anamnesis, and pharmacological therapy was also collected [8, 9]. The mean age at diagnosis was 58.26 years (SD 9.74) [8, 9].

The Movement Disorder Society revised version of the Unified Parkinson’s Disease Rating Scale Part III (18 items, maximum score 72; hereafter called UPDRS) [10] was used to assess clinical motor symptoms. These included language, facial expressions, tremor, rigidity, agility in movements, stability, gait and bradykinesia. Cognitive abilities were tested through an Italian validated version of the Montreal Cognitive Assessment (MoCA) [11]. Cognitive domains assessed include short-term memory (5 points); visuospatial abilities via clock drawing (3 points), and a cube copy task (1 point); executive functioning via an adaptation of Trail Making Test Part B (1 point), phonemic fluency (1 point), and verbal abstraction (2 points); attention, concentration, and working memory via target detection (1 point), serial subtraction (2 points), digits forward and backward (1 point each); language via confrontation naming with low-familiarity animals (3 points), and repetition of complex sentences (2 points); and orientation to time and place (6 points). The total score was given by the sum of these domains, and then divided by the maximum score that could be obtained (30 points). Where one or more domains could not be tested (e.g. visuospatial tasks, due to unavailability of optical devices), the corresponding score was subtracted from the total score obtainable.

Non-motor symptoms were assessed through an Italian validated version of Non Motor Symptoms Scale (NMMS) for Parkinson Disease [12]. This scale tests 9 items, including cardiovascular domain, sleep/fatigue, mood/cognition, perceptual problems/hallucinations, attention/memory, gastrointestinal, urinary, sexual function, and ability to taste or smell. For each item, both severity and frequency of symptoms is measured, so that the scale accounts for both aspects. Here, the sleep domain was slightly modified by adding a further question on the occurrence of vivid dreams. This question was treated as all the others, i.e. the severity of impairment was scored from 0 (no symptoms) to 3 (severe impairment), and the frequency of impairment was scored from 0 (less than once a week) to 4 (daily impairment), then the total score of the sub-item was computed as the product of severity by frequency, and added to the scores of the other sub-items. In the endophenotypes analysis we used UPDRS score < 30 (corresponding to a mild phenotype: 1–2 of Hoehn and Yahr (HY) scale) and ≥ 30 (corresponding to a severe phenotype: 3–5 HY scale) [13], while in non-motor symptoms we used as cutoff ≤54 and > 54 (9 items × 2 (mild impairment) × 3 (weekly impairment)). Statistical significance was set to α = 0.01, correcting for five phenotypes tested.

#### Italian control cohort

As general Italian population, we downloaded whole genome data of 107 samples of the Tuscan Italians (TSI) population of the 1000 Genome Project (phase 3 release) [14] and extracted the exome regions covered by the same capture kit used in our sequencing experiments (SureSelect All Exome kit v6; Agilent® Technologies, Santa Clara, CA, USA).

Moreover, 96 neurological controls, including 38 from MNI and 58 from the Moli-sani genetic biobank (mean age 77 years; SD 5.4; 45 women) [15], underwent targeted resequencing (NGS-TR) of the panel of 26 PD genes identified in this study.

#### International cohort

WES data of 1148 young-onset unrelated PD cases (average age at onset 40.6 years; range 35–56 years) and 503 control participants of European ancestry from the International Parkinson’s Disease Genomics Consortium (IPDGC) were used to replicate the observations made in the Italian cohort. Details on this dataset and on the QC carried out on these samples are reported elsewhere [16].

### Next generation sequencing (NGS)

Genomic DNA was isolated from peripheral blood lymphocytes by PAX gene Blood DNA Midi Kit (QIAGEN, Hilden, Germany).

#### Whole-exome sequencing (WES)

WES was performed on 47 affected individuals (23 families) from PIB cohort, 6 affected individuals (3 families) and 106 independent and unrelated PD cases from MNI cohort. Exonic regions were enriched using the SureSelect All Exome kit v6 (Agilent® Technologies, Santa Clara, CA, USA) based on DNA fragmentation and capture. Exomes were barcoded and sequenced at Helmholtz Zentrum, München, Germany, using the Illumina® HiSeq2000 platform.

#### Targeted resequencing (NGS-TR)

NGS-TR was performed on 288 independent and unrelated PD cases from MNI cohort and on 96 Italian healthy individuals. Probes specific for 100 genes including the 26-targeted genes, identified in this study, and GBA were designed with Nimble Design software (Roche Diagnostics, Mannheim Germany). Targeting regions were enriched using the SeqCap kit based on DNA fragmentation (Kapa Hyper plus kit) and capture (Roche Diagnostics, Mannheim Germany). Targeted regions were barcoded and sequenced on MiSeq platform (Illumina, San Diego, CA, US).

#### Quality control and variant annotation

The alignments of the 100-bp paired-end reads to the human reference genome was performed by using the Burrows Wheeler Aligner (BWA) MEM v0.7.5 [17]. After removal of duplicate reads through Picard MarkDuplicates command (with standard options), we called the single nucleotide variants (SNVs) and insertions/deletions (indels) for all samples using HaplotypeCaller (BP_RESOULTION option) and GenotypeGVCFs in Genome Analysis Toolkit (GATK) v3.5–0-g36282e4, following the manufacturer best practice guidelines (available at https://software.broadinstitute.org/gatk/best-practices/) [18]. Variants with Minor Allele Count (MAC) = 0, number of alternative alleles ≠ 2 and call rate < 95% were also filtered out, as well as samples with identical-by-descent (IBD) sharing and sex mismatches, and samples with call rate < 90%. Similarly, samples were checked for absence of outliers in terms of genome-wide homozygosity, of number of singleton variants and of genetic ancestry (through Multidimensional Scaling Analysis in PLINK) [19]. Variants passing quality control were annotated to genes (within 10 kb from transcription start/stop site) through ANNOVAR [20]. Variant annotation contained information concerning variant type, Minor Allele Frequency (MAF) in the general population, and predictions of the variant’s effect on gene function. MAF was annotated in NHLBI GO Exome Sequencing Project ESP6500si-v2 (European American and African American population), 1000 Genomes Project (AFR [African], AMR [Admixed American], EAS [East Asian], EUR [European], SAS [South Asian], Exome Aggregation Consortium (ExAC) (EUR, non-Finish European population [NFE], AFR, SAS, EAS and AMR). SIFT, PolyPhen2 and Combined Annotation Dependent Depletion (CADD) were used to assess the deleterious effects of the identified variants.

To discover novel PD causative variants we analyzed WES data from 53 PD patients (mean age at onset 61.71, SD 8.57) belonging to 26 late onset PD families with supposedly dominant transmission (Fig. 1), by using a multi-step filtering approach as reported in our previous publications [21, 22]. Prioritization of candidate genes was made through segregation analysis. STRING database analysis was used to identify network of PD-interacting genes products [23].

### Multiple ligation dependent probe amplification (MLPA)

The commercially available kit P051-P052 (MRC-Holland, Amsterdam, Netherlands) was used for the multiplex dosage of exons for the following genes: DJ1 (4 probes in P051), SNCA (5 probes in P051, 1 probe in P052), PARKIN (12 probes in P051, 12 in P052), LRRK2 (8 probes in P052), PINK1 (8 probes in P051). The MLPA was performed on DNA from the 26 families who underwent WES analyses.

#### Polygenic variant load in unrelated Italian individuals

We identified 26 PD candidate genes in this study (*AIMP2, CHMP1A, GIPC1, HMOX2, HSPA8, IMMT, KIF21B, KIF24, MAN2C1, RHOT2, SLC25A39, SPTBN1, TMEM175, TOMM22, TVP23A, ZSCAN21, PARK2, PINK1, DJ-1, LRRK2, HTRA2, FBXO7, EIF4G1, DNAJC6, DNAJC13, SNCAIP*). To assess the co-existence of multiple rare variants in these 26 genes we counted the number of the rare exonic variants in each individual. The protocol used a MAF ≤ 0.001 which was chosen to avoid the loss of heterozygous pathogenic mutations in recessive genes such as *PARK2*, *PINK1* and *DJ1*. The pipeline included:

- Annotation and selection of all the exonic variants;
- Exclusion of synonymous variants;
- Exclusion of variants with alternative allele frequency > 0.001;
- Count of the variants detected per subject in the set of 26 candidate genes.
- Statistical analysis

The co-inheritance of rare variants (MAF ≤ 0.001) was investigated by counting the number of variants laying in the 26 genes, for each sample analyzed, both cases and controls. Fisher’s Exact tests were performed to test differences in the number of cases and controls carrying 0, 1 or ≥ 2 variants. Odds Ratios (ORs) and their corresponding 95% Confidence Intervals (95% CIs) were also estimated in R [24]. Bonferroni correction for two variant load contrasts was applied, resulting in a corrected α = 0.025.

Then an analysis of Receiver Operating Characteristic (ROC) curve was performed to establish the accuracy of the test.

### Expression studies

The expression profile of the 16 novel PD candidate genes *AIMP2*, *CHMP1A, GIPC1*, *HMOX2, HSPA8, IMMT*, *KIF21B, KIF24, MAN2C1, RHOT2*, *SLC25A39*, *SPTBN1*, *TMEM175, TOMM22*, *TVP23A, ZSCAN21* was evaluated on total RNAs from the mesencephalon of adult mice (post-natal day (P) 45). RNA was isolated by using TRIreagent® (Sigma-Aldrich, Saint Louis, MO, USA) protocol. 2 μgs of total RNA were reverse transcribed with the Superscript III-First strand kit (Thermo Fisher Scientific, Waltham, MA, USA). Quantitative PCR (qPCR) reactions were performed in triplicate, using gene specific primers (Table S2) and ITaq Universal Sybr Green Supermix (Bio-Rad, Hercules, CA, USA) following the manufacturer’s directions. Results were normalized versus the expression of the glyceraldehydes-3-phosphate dehydrogenase (*Gapdh*) gene. *Pitx3* was used as an example of mdDA neurons restricted gene expression marker. Standard deviation was calculated by using data of three different experiments.

### Immunohistochemistry assays

Immunohistochemistry was performed on paraformaldehyde-fixed, wax-included brains as described previously [25]. Mouse and rat adult brains were obtained from healthy animals sacrificed in accordance with the recommendations of the European Commission. All the procedures related to animal treatments were approved by Ethic-Scientific Committee for Animal Experiments and Italian Health Ministry. Formalin-fixed and paraffin-embedded (FFPE) Substantia Nigra tissue sections from human adult normal brain were purchased at BioChain (Newark, CA, USA). Rabbit antibodies were directed against SLC25A39 (Abcam, Cambridge, UK, Ab-105,683; dilution 1:80) and Tyrosine Hydroxylase (TH) (Chemicon International, Temecula, CA, USA, ab152; dilution 1:600). Mouse antibodies were directed against GIPC1 (Santa Cruz Biotechnology, Dallas, TX, USA, Sc-271,822; dilution 1:80), TOMM22 (Abcam, Cambridge, UK, Ab-57,523; dilution 1:80), ZSCAN21 (Novus Biologicals, Centennial, CO, USA, NBP2–45443; dilution 1:80), HSPA8 (Santa Cruz Biotechnology, Dallas, TX, USA, Sc-59,570; 1:100), TH (Chemicon International, Temecula, CA, USA, MAB318; dilution 1:500).

### URLs

STRING (https://string-db.org/); 1000 Genomes Project phase 3 release (ftp://ftp.1000genomes.ebi.ac.uk/vol1/ftp/release/20130502/); Human Gene Mutation Database (HGMD, http://www.hgmd.cf.ac.uk/ac/index.php; Jun-2017 version); Parkinson Disease Mutation Database (PDmutDB, https://www.molgen.vib-ua.be/PDMutDB/; Jun-2017 version); NHLBI GO Exome Sequencing Project ESP6500si-v2 (http://evs.gs.washington.edu/EVS/); 1000 Genomes Project (http://www.1000genomes.org/); Exome Aggregation Consortium (ExAC) (http://exac.broadinstitute.org/); SIFT (http://sift.bii.a-star.edu.sg/www/SIFT_seq_submit2.html/); PolyPhen2 (http://genetics.bwh.harvard.edu/pph2/); Combined Annotation Dependent Depletion (CADD) (cadd.gs.washington.edu/); R (http://www.R-project.org/).

## Results

### Identification of novel candidate genes for Parkinson’s disease

The study aimed at the identification of genetic risk profiles for PD. The discovery cohort included 53 PD patients belonging to 26 families showing a dominant mode of inheritance of the disease (Fig. 1). As validation cohort, we used 1542 independent PD cases and 706 controls from different data sets of European ancestry (see Patients and Methods for details) (Table S1) [8, 9, 16, 26].

We first analysed the WES data (without applying any filter) of the 53 PD patients belonging to 26 families to search for mutations in genes already known to be associated to dominant form of PD such as, *SNCA, VPS35* and *LRRK2*. Subsequently, we searched for large insertion/deletions in *SNCA* and *LRRK2* genes by using MLPA approach. All annotated variants were surveyed into the Parkinson’s disease Mutation Database (https://grenada.lumc.nl/LOVD2/TPI/home.php) to confirm their pathogenicity. One out of the 26 analyzed families carried a pathogenic mutation in *LRRK2* gene (c.G4322A, p.R1441H).

**Fig. 2:**
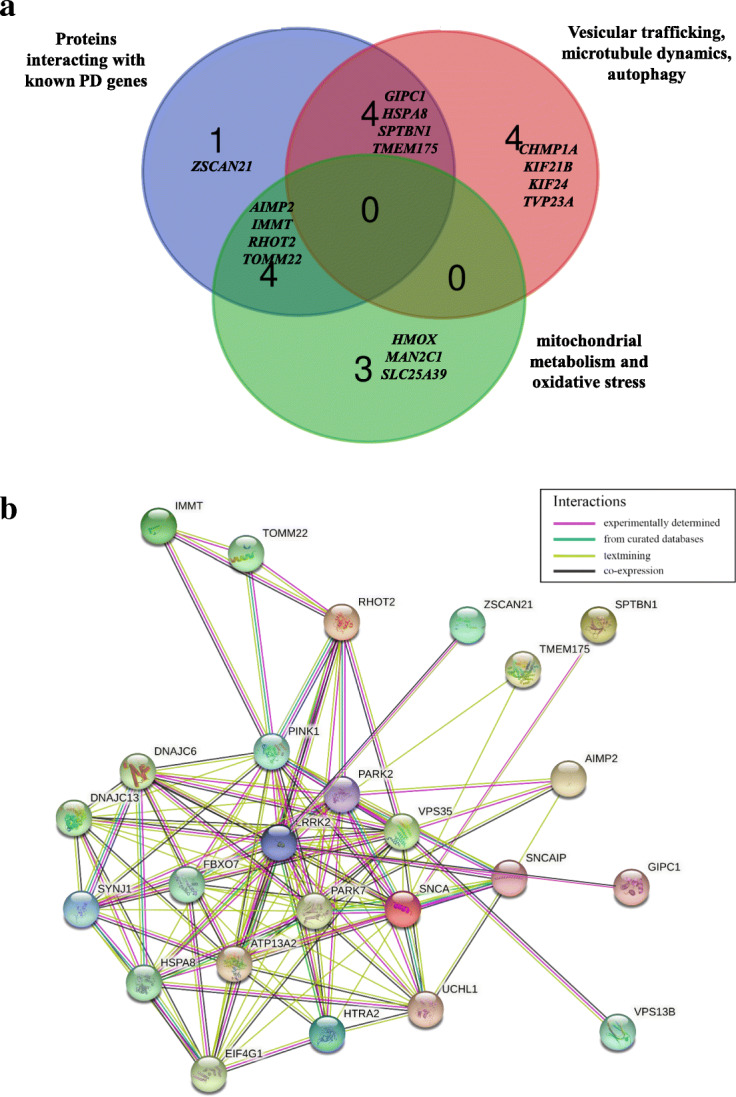
Venn diagram and STRING analysis representing the logical relations between the 16 novel gene products and the network of PD-interacting genes products. **a** Venn diagram showing the 16 novel identified genes involved across different interconnected pathways: interactors of PD related genes, genes involved in mitochondrial metabolism and those involved in vesicular trafficking and autophagy. **b** Schematic representation of the network of interactions of the 16 novel PD gene products with known PD-associated genes. STRING database analysis identified 9 genes interacting with PD-associated genes

To search for novel candidate-disease genes we used a multistep filtering approach. We selected splicing variants (regardless of their exonic or intronic position; minor allele frequency (MAF) ≤ 0.001) and exonic disruptive variants (MAF ≤ 0.001; CADD phred score ≥ 20; excluding synonymous changes), shared between affected relatives within each of the 26 families (siblings or parent-offspring pairs), assuming a dominant mode of transmission of the disease. The variants were selected assuming a MAF ≤ 0.001 in public exome databases including ESP6500si-v2, 1000 Genomes Project and ExAC. The cut off was set to 0.001, taking in account the prevalence of rare dominant diseases (1–2/10,000) and the MAF of the most frequent mutation associated with late onset PD p.(G2019S) in the *LRRK2* gene, also known as rs34637584, which shows a MAF ranging from 0.001 to 0.0002 in different databases. Retained variants (6488) were filtered for CADD phred score ≥ 20 (3712 remaining variants), corresponding to those variants which are predicted to be amongst the 1% most deleterious variants in the genome. Subsequently we compared data of the affected relatives of each family to select shared variants. This analysis identified an average number of 10 variants shared between PD relatives per family. Segregation analysis was performed in additional affected relatives belonging to the same 26 PD families (1–3 individuals per family based on DNA availability) (Fig. 1). Considering the late age at onset of the disease unaffected family members were not investigated. This analysis disclosed 28 rare disruptive variants (23 non-synonymous, 2 stop-gain, 1 frameshift, 2 non-frameshift deletions) laying in 26 genes, which were shared among familial PD cases in 18 out of the 26 analyzed families (Table 1). Among these, in 10 families we found single heterozygous deleterious variants in a single gene segregating with PD phenotype, supporting a dominant model of inheritance. Instead, we identified 2 variants in 6 families and 3 variants in 2 families in different genes segregating with PD phenotype suggesting a polygenic model of inheritance. Unfortunately, in the remaining eight families we were not able to identify potential causative variants, probably due to the highly stringent criteria used in the pipeline of analysis. Seven (*PARK2, PINK1, DJ-1(PARK7), LRRK2, FBXO7, DNAJC6, DNAJC13)* out of the 26 identified genes were already known as causing PD, including familial recessive forms, while three (*HTRA2, EIF4G1, SNCAIP)* genes were PD candidate genes/at risk factors for which a clear link with the disease is not completely established [5, 6]. Moreover, it is interesting to note that in several families, single mutations in recessive genes such as *PARK2*, *PINK1*, *PARK7*, were co-inherited with variants in other candidate genes, suggesting that they might play a role as risk factors in the heterozygous status. The presence of a second mutation in these recessive genes including large deletions/insertions was excluded trough MLPA approach. Sixteen out of the 26 genes analyzed were novel PD candidate genes involved in pathways potentially deregulated in PD such as mitochondrial metabolism and oxidative stress (*AIMP2*, *HMOX2, IMMT, MAN2C1, RHOT2, SLC25A39, TOMM22*) [27–35], vesicular trafficking, microtubule dynamics, autophagy (*CHMP1A, GIPC1*, *HSPA8*, *KIF21B, KIF24, SPTBN1, TMEM175, TVP23A*) [36–42] or in *SNCA* gene expression (ZSCAN21) (Fig. 2a) [43, 44]. STRING database analysis showed that nine out of the 16 novel genes (*AIMP2*, *GIPC1, HSPA8*, *IMMT*, *RHOT2*, *SPTBN1, TMEM175, TOMM22*, *ZSCAN21*) encoded for proteins interacting with known PD genes (Fig. 2a and b) [39, 45].

**Table 1:** Rare disruptive variants (MAF ≤ 0.001, CADD phred≥20) in the 26 PD candidate genes identified in the discovery families

CHR	Genomic position (hg19)	dbSNP	Gene	RefSEq	Nucleotide Change	AA Change	Exonic Function	Cl	MAF Max in public data sets	CADD phred	Family
16	723,544	rs143816083	RHOT2	NM_138769	c.G1795T	p.G599W	NSV	N	0.0001	25	1
3	184,039,223	rs746291399	EIF4G1	NM_198241	c.C851G	p.S284C	NSV	N	0.00004	25	2
1	8,022,928	rs142405016	PARK7	NM_007262	c.G83A	p.R28Q	NSV		0.0002	35	5
7	6,062,997	rs749728733	AIMP2	NM_006303	c.T638C	p.I213T	NSV	N	0.00002	27	7
2	86,408,450	rs201861204	IMMT	NM_001100169	c.C91T	p.R31C	NSV	N	0.0002	26	7
1	20,971,042	rs74315358	PINK1	NM_032409	c.G836A	p.R279H	NSV	P	0.00004	26	7
16	89,717,990		CHMP1A	NM_002768	c.C92T	p.A31V	NSV	N	NA	23	9
2	74,757,823		HTRA2	NM_013247	c.A586G	p.N196D	NSV	N	NA	23	9
3	132,222,165		DNAJC13	NM_015268	c.C4824G	p.S1608R	NSV	N	NA	20	10
17	42,398,033		SLC25A39	NM_001143780	c.C758A	p.T253N	NSV	N	NA	21	10
2	54,895,594	rs200093475	SPTBN1	NM_003128	c.C6983A	p.T2328N	NSV	N	0.00004	27	12
22	39,078,021	rs200476832	TOMM22	NM_020243	c.C38T	p.P13L	NSV	N	0.0002	25	12
19	14,589,354		GIPC1	NM_202470	c.875dupA	p.K292fs	Fs_ins	N	NA	NA	13
6	162,394,354	rs114974496	PARK2	NM_004562	c.C714G	p.C238W	NSV	P	0.00004	25	13
5	121,787,252	rs751412863	SNCAIP	NM_005460	c.A2710G	p.K904E	NSV	N	0.00004	24	14
12	40,704,237	rs34995376	LRRK2	NM_198578	c.G4322A	p.R1441H	NSV	P	0.00001	27	14
7	99,654,828		ZSCAN21	NM_145914	c.C199T	p.R67W	NSV	N	NA	24	16
9	34,290,328	rs372797268	KIF24	NM_194313	c.T971C	p.I324T	NSV	N	0.0001	26	17
16	10,911,991		TVP23A	NM_001079512	c.G58T	p.E20X	stopgain	N	NA	38	18
1	65,858,474		DNAJC6	NM_001256864	c.C1829T	p.P610L	NSV	N	NA	23	19
15	75,652,021		MAN2C1	NM_001256494	c.1882_1887del	p.628_629del	NFs_del	N	NA	NA	19
15	75,652,016		MAN2C1	NM_001256494	c.1890_1892del	p.630_631del	NFs_del	N	NA	NA	19
22	32,870,999		FBXO7	NM_012179	c.C10T	p.R4W	NSV	N	NA	33	20
16	4,557,822	rs772245870	HMOX2	NM_001286271	c.C226T	p.R76W	NSV	N	0.00001	34	20
1	200,957,957	rs751952433	KIF21B	NM_001252100	c.G3235A	p.A1079T	NSV	N	0.00002	29	21
11	122,931,465		HSPA8	NM_006597	c.G247A	p.V83I	NSV	N	NA	21	24
4	947,062		TMEM175	NM_032326	c.C547T	p.R183X	stopgain	N	NA	34	25
1	200,974,537		KIF21B	NM_001252100	c.C631T	p.R211C	NSV	N	NA	34	26

*CHR* Chromosome, *hg19* human genome build to which these variants are annotated, *dbSNP* reference number in SNP database, *ref. seq* reference number of the gene transcript, *AA Change* Amino acid change, *CI* Clinical interpretation, *P* Pathogenic, *UP* Uncertain pathogenicity, *N* Novel, *PD* Parkinson’s disease, *IPDGC* International Parkinson’s Disease Genetics Consortium, *CADD phred* Combined Annotation Dependent Depletion, *Fs_ins* Frame shift insertion, *NFs_del* Non Frame shift deletion, *NSV* Non-synonymous variant, *MAF* Minor Allele Frequency, *NA* Not Annotated, *MAF* max in public datasets: highest allelic frequency annotated in public databases including 1000 Genomes Project (AFR. AMR. EAS. EUR. SAS), ExAC browser (NFE. AFR. SAS. EAS and AMR), ESP6500si-v2 (European American and African American population)

Only very recently, *IMMT*, *ZSCAN21* and *TMEM175* have been associated to PD [43, 46, 47] further supporting the discovery pipeline employed in this study.

To further support the genetic involvement of the 16 novel identified genes in PD pathogenesis, we extended the mutational analysis to 1542 independent PD patients (including MNI (394 cases) and IPDGC (1148 cases)) and 706 controls from different cohorts of European ancestry, to detect further disruptive variants within these genes and to compare the total load of these variants between cases and controls. Overall data identified 256 different variants (MAF ≤ 0.001; CADD phred score ≥ 20), of which 170 were present only in cases, 61 only in controls and 25 were shared between cases and controls. Remarkably, the 170 different deleterious variants (153 non-synonymous, 5 stop-gain, 6 frameshifts, 3 non-frameshift deletions and 3 splicing) were identified in 90 Italian patients (MNI cohort) and 153 patients of the IPDGC cohort, confirming the extreme genetic heterogeneity at the basis of PD (Table S3). None of these variants was found in 706 healthy control subjects, including both IPDGC and Italian population controls (namely MNI healthy subjects and Tuscans (TSI) “pseudo-controls “of the 1000 Genomes Project) [14]. Interestingly, significant enrichment of variants in these 16 genes was observed in patients compared to controls (243 patients (15.7%) vs 69 controls (9.7%); OR = 1.73 [1.3–2.29]; *p* = 0.0001 χ ^2^ = 14.01).

**Fig. 3:**
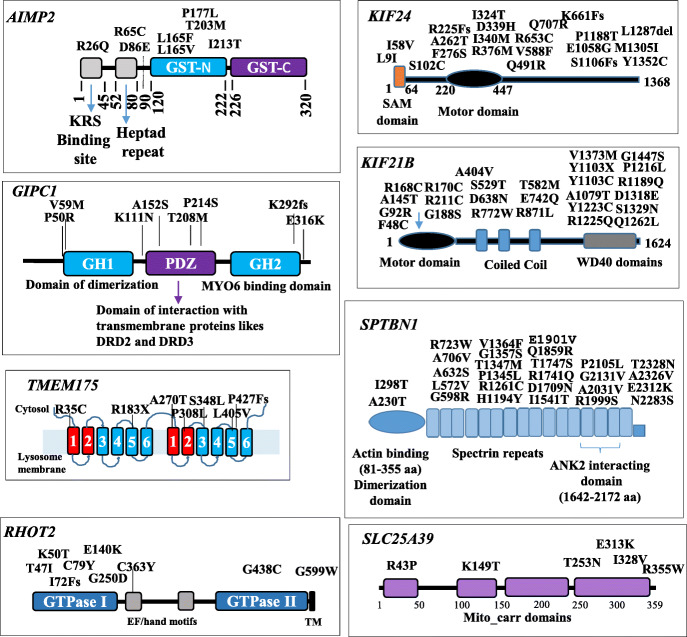
Graphical representation of the protein domains and mapped mutations. For those genes whose protein structure was available (*AIMP2, GIPC1, KIF24, KIF21B, RHOT2, SLC25A39, SPTBN1, TMEN175*), we report the protein domains and mapped amino acid changes identified in the cohort of 1542 patients. Amino acid changes are referred to by their single letter code

Noteworthy, all the affected residues identified in PD patients occupy functionally important amino acid positions, which are highly conserved amongst vertebrates (Fig. 3; Fig. S1). In summary, although functional data are necessary to substantiate the causative role of the variants identified, these results suggest a potential involvement of the candidate genes in PD etiopathogenesis and indicate an extreme genetic heterogeneity of mutations in PD patients.

#### Expression analysis in human, mouse and rat DA neurons

**Fig. 4:**
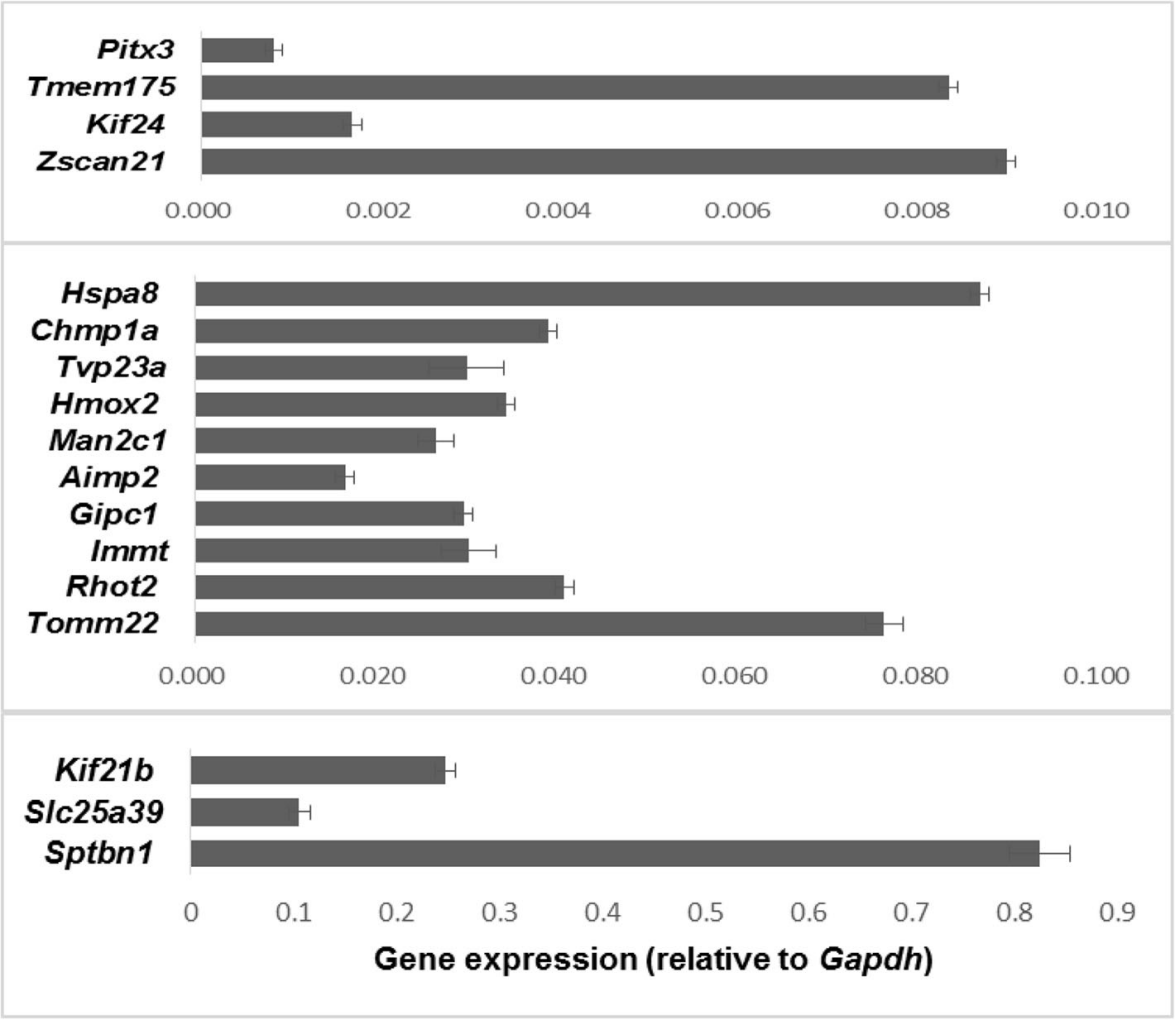
Expression analysis of the novel identified Parkinson’s disease genes. qPCR experiment showed that the 16 novel genes were expressed in mesencephalon of P45 adult mice. *Pitx3* was used as an example of mdDA neurons restricted gene expression. *Gapdh* was used to normalize the results. Data were shown as absolute values with standard deviation calculated from three different experiments. Genes were grouped on the basis of their expression level in three different scales

**Fig. 5:**
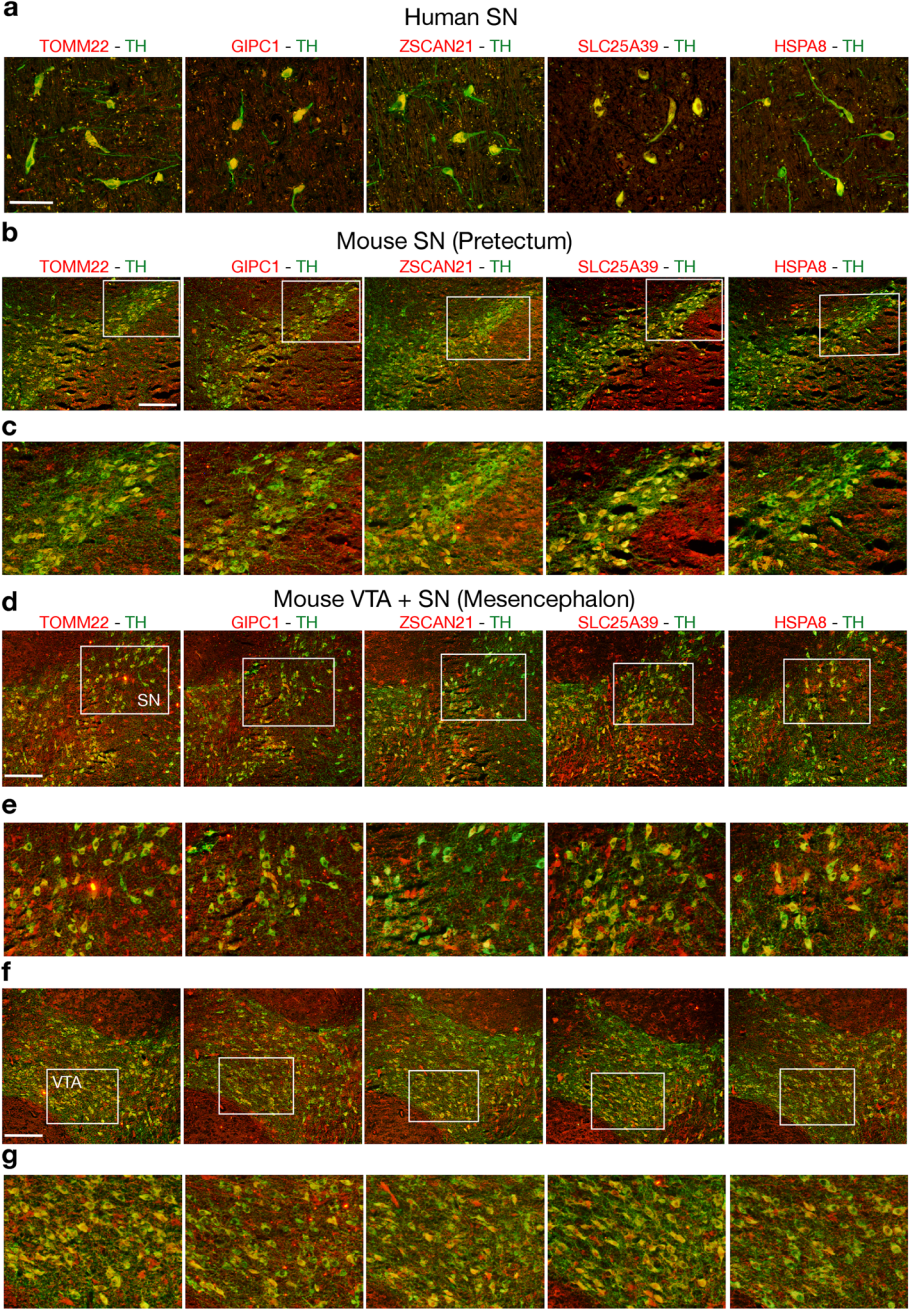
Expression analysis of TOMM22, GIPC1, ZSCAN21, SLC25A39 and HSPA8 in human and mouse DA neurons. **a-g** Immunohistochemistry experiments performed on human (**a**) and P45 mouse (**b-g**) adult brain sections showed that TOMM22, GIPC1, ZSCAN21, SLC25A39 and HSPA8 were co-expressed with TH in DA neurons of the human SN (**a**) and in mouse DA neurons of the SN (**b-e**) and VTA (**f, g**). Images in (**c, e, g**) correspond to magnification of the boxed area reported in (**b, d, f**). Scale bars correspond to 100 μm. Abbreviations: SN stands for substantia nigra and VTA for ventral tegmental area

Expression analysis through quantitative PCR (qPCR) assays showed that the 16 novel PD genes were all transcribed in the mesencephalon of adult mice at post-natal day (P) 45 (Fig. 4). Then we studied through immunohistochemistry experiments whether five representative genes (TOMM22, GIPC1, ZSCAN21, SLC25A39 and HSPA8) were co-expressed with TH in adult DA neurons of the substantia nigra (SN) and ventral tegmental area (VTA). We first analyzed adult human SN neurons and we found that TH^+^ neurons co-expressed all the five genes (Fig. 5a and Fig. S2a). This analysis was repeated in P45 mouse (Fig. 5b-g and Fig. S2b-d) and P60 rat (Fig. S3) brains. In mouse mdDA neurons belonging to the anterior SN located in the pretectum (Fig. 5b, c and Fig. S2b) or to the SN and VTA of mesencephalic origin (Fig. 5d-g and Fig. S2c, d) showed that the expression of TOMM22, GIPC1, ZSCAN21, SLC25A39 and HSPA8 colocalized with most of the TH^+^ neurons (Fig. 5b-g and Fig. S2b-d). A similar result was observed when this expression analysis was performed in rat SN and VTA neurons of mesencephalic origin (Fig. S3). These data indicate that at least five out of the 16 novel PD genes were expressed in mdDA neurons supporting the possibility that they may be cell-autonomously involved to provide mdDA neurons with functional features potentially controlling identity and/or survival and/or DA neurotransmission. Therefore, together with previous data, this expression analysis further supports the possibility that mutations identified in the 16 novel PD candidate genes may potentially predispose for PD occurrence.

#### Polygenic load analysis of rare exonic variants in familial and sporadic cases

**Fig. 6:**
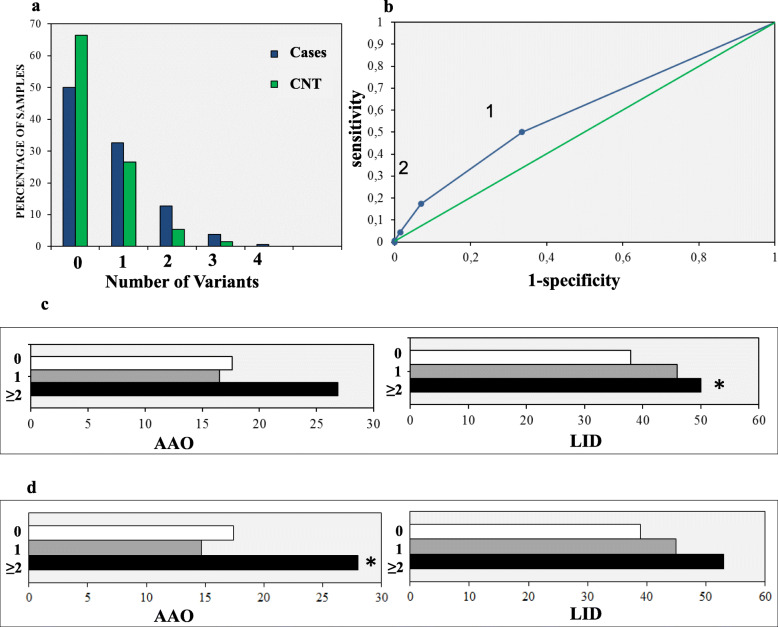
Distribution of variants load, ROC curve and analysis of PD endophenotypes. **a, b** Very rare (MAF ≤ 0.001), exonic variants (exclusion of synonymous changes) were annotated in 26 PD genes in 394 unrelated Italian cases and in 203 controls. **a** The histogram shows the percentage of samples (cases in blue and controls in green) carrying 0, 1, 2, 3, 4 variants in the selected genes; **b** ROC curve and analysis of sensitivity and specificity. The test shows that the distribution is high significant and the test may predict the disease in about 17% of at risk individuals in the general population, carrying at least 2 variants, with specificity > 93%. **c** Percentage of PD cases manifesting L-dopa-induced dyskinesia (LID), and earlier age at onset (AAO) of Parkinson’s disease. **d** Polygenic variant load, including *GBA* variants, was inversely associated with age at PD onset at the nominal significance level (p 0.044). For each comparison, we set statistical significance threshold at *p* < 0.05. Statistical significance was reported with asterisk

Based on the observation that 8 out of the 18 families of the discovery cohort carried two or more variants in the 26 PD genes (Table 1), we hypothesized that the genetic combination of multiple rare variants in these genes may represent a specific genetic hallmark of PD risk. To test this hypothesis, we analyzed in 394 unrelated and independent PD patients (including 172 familial and 222 sporadic cases from MNI data set) and in 203 controls (MNI, Moli-sani, and TSI data sets) [8, 9, 14, 15] the co-inheritance (per individual) of rare (MAF < 0.001) exonic (excluding synonymous) and splicing variants detected in the panel of the 26 genes (details in Patients and Methods). For this analysis, we selected rare variants based on a frequency threshold (MAF ≤ 0.001), without considering their degree of deleteriousness. This analysis identified 220 different variants among cases and 65 in the controls; only 18 of them were shared between case and controls (Table S4). The co-inheritance of these variants is reported in Table 2 and Fig. 6a. We observed that, approximately 17% of the PD patients carried two or more variants (cases 17.3% vs controls 6.8%; OR = 3.3 [1.8–6.7]; *p* = 4.4 × 10^− 5^) (Table 2). Remarkably, sporadic cases showed a significant distribution within the same class (sporadic cases 13.9% vs controls 6.8%, OR = 2.6 [1.3–5.1]; *p* = 0.005) (Table 2). These differences remained statistically significant after Bonferroni correction for multiple testing of two contrasts (0 vs 1 and 0 vs ≥2 variants load classes) (α = 0.025). This finding corroborates the polygenic view of PD risk for both sporadic and familial cases. To measure the accuracy of the test, we applied the ROC curve and found a maximum sensitivity of 0.50 (based on the presence of at least one variant (specificity 0.66)) and a maximum specificity > 0.93 (based on the co-occurrence of two or more variants (sensitivity of 0.17)) (AUC = 0.59, SE = ±0.020, *p* = 4.42 × 10^− 6^) (Fig. 6b). Overall, the presence of two or more disruptive variants in the 26 candidate genes represented the best tradeoff between sensitivity (0.17) and specificity (0.93). Moreover, considering that is well established that rare variants in *LRRK2*, *PINK1*, *PARK2* and *PARK7* genes have a higher prevalence in PD patients compared to healthy individuals [48–52], we analyzed the presence of at least one rare variant (MAF < 0.001) in patients and controls taking into account solely the 16 novel candidate genes. This analysis confirmed an excess of patients carrying rare variants in the 16 novel genes (cases 31.6% vs controls 23.1%; *p* = 0.014), as also reported in the gene identification paragraph for rare deleterious variants (MAF ≤ 0.001; CADD phred score ≥ 20) selected in the 16 novel genes in cases and controls (243 (15.7%) vs 69 (9.7%); OR = 1.73 [1.3–2.29]; *p* = 0.0001 χ ^2^ = 14.01), further supporting their role into PD etiopathogenesis.

**Table 2:** Polygenic variant load in the Italian cohort

Series	N variants	PD cases (***n*** = 394)	FPD (***n*** = 172)	SPD (***n*** = 222)	Cnt. (***n*** = 203)	p	OR	95%CI
**MAF ≤ 0.001**	0	197 (50.0%)	82 (47.6%)	115 (51.8%)	135 (66.5%)	baseline		
	1	129 (32.7%)	53 (30.8%)	76 (34.2%)	54 (26.6%)	0.014	1.6	1.1–2.4
	≥2	68 (17.3%)	37 (21.6%)	31 (13.9%)	14 (6.8%)	4.4 × 10^−5^	3.3	1.8–6.7
**MAF ≤ 0.001** **including variants in** ***GBA*** **gene**	0	182 (46.2%)	79 (45.9%)	103 (46.4%)	128 (63.0%)	baseline		
	1	130 (33.2%)	53 (30.8%)	77 (35.1%)	59 (29.0%)	0.028	1.55	1.04–2.32
	≥2	82 (20.5%)	40 (23.2%)	42 (18.5%)	16 (7.2%)	3.4 × 10^−6^	3.59	1.97–6.90

*MAF* Minor allele frequency, *PD* Parkinson’s disease, *FPD* Familial PD cases, *SPD* Sporadic PD cases, *Cnt* Controls fromTSI, Tuscan Italian population, MNI cohort and Moli-sani genetic biobank, *p p*-value calculated with Fisher Exact Probability Test, *OR* Odds Ratio, *CI* Confidence interval

Moreover, the pool of rare variants identified in the PD cases was different from those detected in controls (Table S4). This suggests that, besides the load of rare variants (≥2), also the nature of the mutation is important in predisposing/causing PD.

Thus, the availability of a detailed database including variants detected only in PD cases or only in controls might be relevant to improve the operational characteristics of our predictive protocol in the general population.

#### Analysis of *GBA* gene

To test the involvement of *GBA* gene, that is the most important genetic risk factor for PD, alone and in combination with the 26 PD genes, we analyzed all *GBA* exonic variants annotated in the 26 late onset PD families and in the Italian validation cohort (394 independent PD cases and 203 controls) (Table S5). In the discovery cohort the *GBA* variant c.G1093A p.E365K (E326K, previous nomenclature) was present in the two affected siblings of family 7, co-inherited with single heterozygous variants in *AIMP2*, *IMMT* and *PINK1* genes (Table 1), while the *GBA* variant c.C1223T p.T408M (T369M, previous nomenclature) was present in one of the two siblings of family 16 who also carried the c.G4322A p.R1441H mutation in *LRRK2* gene and the deleterious variant c.A2710G p.K904E in *SNCAIP*. In the independent cohort of PD cases and controls we found a significant distribution of *GBA* variants (42 cases (10.6%) vs 8 controls (3.9%); *p* = 0.002, OR = 2.91 [1.34–6.32]).

Polygenic load analysis including multiple rare variants in the 26 genes as well as rare pathogenic variants in *GBA* gene showed that, approximately 20% of the PD patients carried two or more variants (cases 20.5% vs controls 7.2%; OR = 3.59 [1.97–6.90]; *p* = 3.4 × 10^− 6^) (Table 2). Remarkably, the increase in significance was mainly attributable to sporadic cases (sporadic cases increase from 13.9 to 18.5% with GBA mutations) (Table 2).

#### Analysis of PD endophenotypes

To investigate the relation of the polygenic variant load in the selected 26 candidate genes with PD endophenotypes, including both motor and non-motor symptoms, we analyzed the cohort of 394 PD-MNI unrelated patients mentioned before, for which clinical assessments were available (see Patients and Methods for details on the scales used). More specifically, we tested how the presence of 0, 1 ≥ 2 rare variants in the 26 PD genes affected PD age at onset (AAO), motor symptoms (UPDRS scale) [10, 13], non-motor symptoms (NMS scales) [12] and presence of L-dopa induced Dyskinesia (LID), a common motor side effect of levodopa therapy in PD patients [8, 9]. Overall data show that the selected genes might influence preferentially LID occurrence, although the contrast would not survive correction for multiple testing of five phenotypes (*p* 0.038; Fig. 6c; Table S6A). When we took into account also GBA variants, this contrast was not significant anymore, while variant load was inversely associated with age at PD onset at the nominal significance level (p 0.044; Table S6B; Fig. 6d).

## Discussion

In this study, we discovered a set of 16 genes worthy of future studies to confirm their role in onset or progression in PD. We identified 170 rare and deleterious variants, absent in healthy individuals, in 15.7% of PD patients (243 PD cases out of 1542) supporting the extreme genetic heterogeneity of mutations in PD.

Although additional studies are needed to confirm the functional role of the novel identified genes in PD etiopathogenesis, a number of published studies support this hypothesis.

In particular, the transcription factor ZSCAN21 may directly control *SNCA* gene expression [28, 37], and the c.C199T p.(R67W) mutation identified in this study affects a highly conserved arginine residue necessary for the formation of the ZSCAN21 functional homodimer [53]. TMEM175 is a lysosomal K+ channel that is required to maintain membrane potential and pH stability in lysosomes as well as to control α-synuclein aggregation [42, 54]. These functions may be affected by knock down of the gene *TMEM175* [42]. Although *TMEM175* was previously identified in a genome wide association study of PD [47], detrimental mutations have not been identified before this study.

RHOT2 (MIRO2), IMMT and TOMM22 directly interact with PINK-1 and Parkin and influence both mitochondrial homeostasis and vulnerability of DA cells to toxins [27–31, 55]. The aminoacyl-tRNA synthetase complex interacting multifunctional protein-2 (AIMP2) is a parkin substrate and its overexpression leads to a selective, age-dependent, progressive loss of DA neurons via activation of poly (ADP-ribose) polymerase-1 (PARP1) [35]. GIPC1 interacts with LRRK2 in DA cells in vitro and in the mouse ventral mesencephalon in vivo [45]. The Drosophila homolog of *GIPC1* is expressed in the central brain of adult flies and its reduced expression significantly affects locomotive activity and longevity [56]. SLC25A39 is a mitochondrial carrier of manganese that promotes neuronal survival in Drosophila [34]. βII-spectrin, encoded by *SPTBN1*, is required for axon growth and axonal transport of synaptic cargo [57]. Moreover, it specifically interacts with α-synuclein and its overexpression induces neurite formation [39]. The chaperone-mediated autophagy (CMA) protein HSPA8/HSC70 (heat shock protein 8) is essential in post-mitotic neurons for diluting toxic intracellular components including the disassembling of α-synuclein amyloid fibrils [58]. KIF21B is a processive kinesin whose depletion alters neuronal dendritic tree branching and spine formation [37]. Noteworthy, similarly to *Kif21b* knockout mice, which exhibit learning and memory deficits [37], our patients carrying *KIF21B* mutations (Table S3) showed mild cognitive impairment assessed with Montreal Cognitive Assessment (MoCA) test (mean 23 SD 2). KIF24 is a centriolar kinesin that specifically regulates ciliogenesis by dynamically controlling microtubule polymerization [38]. Single Nucleotide Polymorphisms (SNPs) located in the *KIF24* gene have been associated to Frontotemporal Lobar Degeneration (FTLD) [59]. Interestingly, the patients of the MNI cohort carrying mutations affecting the motor domain of KIF24 protein (Fig. 3) showed mild-severe cognitive impairment (MoCA score ranging from 13 to 23).

HMOX exerts both pro- and anti-oxidant effects, which may influence the pathogenesis or progression of Parkinson’s disease [32, 60]. α-Mannosidase (MAN2C1) is the enzyme responsible for the partial demannosylation occurring in the cytosol. *Man2c1*-deficient mice revealed neuronal and glial degeneration [61]. Charged multivesicular body protein 1A (CHMP1A) is a member of the ESCRT-III (endosomal sorting complex required for transport-III) complex which localizes to the nuclear matrix and regulates chromatin structure [36, 62]. CHMP1A regulates proliferation of neuronal progenitor cells [36, 62]. *TVP23A* is a novel gene reported in gene database (Gene ID: 780776) as involved in intracellular vesicular transport.

Previous studies identified the p.G2019S mutation in *LRRK2* as a major causative event in 1–4% of PD patients, depending on the analyzed population [63]. Within our cohort of patients, the p.G2019S mutation in *LRRK2* was detected in 8 out of 394 PD patients (2%). Moreover, other studies also correlated the severity but not the occurrence of the PD to the cumulative effect of *LRRK2* mutations and rare variants in other PD related genes (e.g. GBA) [26, 64–66].

In this study, we highlight the polygenic nature of both sporadic and familial forms of PD, especially when the familial forms come from small nuclear families. We identified a panel of 26 candidate genes carrying likely pathogenic variants and observed the presence of multiple rare variants (≥ 2) in these 26 genes in about 17% of PD patients, including familial and sporadic cases. This percentage increased to 20% of PD patients when we included rare pathogenic variants in *GBA.* Remarkably, this increase was mainly attributable to sporadic cases.

This means that the molecular diagnosis of PD should always take in account the analysis of multiple candidate genes. However, although the evidence reported here looks very promising, at this stage of advancement of our study we cannot offer any improvement in practicing medicine, until further confirmatory studies and functional validations are warranted. Undoubtedly, the extreme genetic heterogeneity observed in PD patients, everyone genetically and biologically unique, carrying different genetic combinations of rare variants not shared by the majority of PD patients, could cause great uncertainty in the interpretation of the results. Nevertheless, at a molecular level, these findings offer a rational support to address additional genetic studies testing this protocol in larger cohort of PD patients and controls. If these data were confirmed we could consider the possibility of using this protocol to identify, with high specificity, subjects potentially at risk of developing PD by counting per individual the number of rare variants in the panel of candidate genes.

Moreover, our data also underline the importance of rare variants in the genetics of sporadic PD and corroborate the proposed approach as complementary to genome wide association studies (GWAS) and to classical co-segregation analyses of NGS data to identify variants implicated in PD.

The present study also reports, for the first time, statistical evidence that the polygenic variant load in the PD candidate genes analyzed is associated with the occurrence of L-dopa induced Dyskinesia, an important motor side effect of PD treatment. Although it is tempting to speculate about the translational relevance of this finding, caution is suggested in the interpretation since the association was only nominally significant and disappeared when GBA variants were included in the analysis. Interestingly, in the latter setting a high variant burden was associated with earlier PD onset, in line with previous evidence [67, 68].

## Conclusions

Our findings encourage further studies to improve the predictive/diagnostic power of this protocol for PD and lead us to surmise that this strategy may hypothetically be extended to other polygenic disorders with largely unknown genetic etiology, such as dyslexia [69].

Therefore, in the future studies on PD genetic aetiology should be directed towards analyzing the polygenic burden of rare variants with presumably large effect size, and validating them at the functional level.

## Supplementary Information

**Additional file 1: Figure 1S.** Evolutionary conservation study of the likely pathogenic variants identified in the 16 novel Parkinson’s disease candidate genes. The affected residues are reported in bold underlined. **Figure 2S.** Single channel images of human and mouse immunostainings shown in Fig. 5. (**a-d**) Single channel images showing expression of TH in combination with TOMM22, GIPC1, ZSCAN21, SLC25A39 and HSPA8 in human SN neurons **(a)** and in pretectal SN **(b)** mesencephalic SN **(c)** and VTA **(d)** mouse neurons. Images in **(a)** correspond to those reported in Fig. 5a; images in **(b-d)** correspond to those reported in Fig. 5c, e, g, respectively. **Figure 3S.** Expression analysis of TOMM22, GIPC1, ZSCAN21, SLC25A39 and HSPA8 in rat DA neurons. **a-e** Immunohistochemistry experiments performed on P60 rat brain sections showed that TOMM22, GIPC1, ZSCAN21, SLC25A39 and HSPA8 were co-expressed with TH in mesencephalic DA neurons of the SN (**b, c**) and VTA (**d, e**). Images in (**c, e**) correspond to magnification of the boxed area reported in (**b, d**). Sections in (**a**) are low magnification images showing only TH immunostaining and corresponding to the same sections reported in (**b-e**). Scale bars correspond to 100 μm. Abbreviations: SN stands for substantia nigra and VTA for ventral tegmental area. In (**c, e**) are shown merge and single channel images. **Table S1.** Cohort description. **Table S2.** List of primers used for gene expression analysis by quantitative PCR (qPCR). **Table S3.** Rare disruptive variants (MAF ≤ 0.001, CADD phred ≥ 20) identified in the 16 novel PD candidate genes in the validation cohorts. **Table S4.** Rare variants (MAF ≤ 0.001) in the 26 PD candidate genes in affected and healthy individuals of the Italian cohort. **Table S5.** GBA variants annotated in Italian cohort of patients and controls.

## Data Availability

The datasets used and/or analyzed during the current study are available from the corresponding author on reasonable request.

## Abbreviations

PD: Parkinson’s disease
LOPD: Late onset Parkinson’s disease
GWAS: Genome wide association studies
mdDA: Mesencephalic-diencephalic DA
SNpc: Substantia nigra pars compacta
PIB: Parkinson Institute Biobank
WES: Whole exome sequencing
MNI: Mediterranean Neurological Institute
NGS: Next Generation Sequencing
TR: Targeted Re-sequencing
SNVs: Single nucleotide variants
MAF: Minor Allele Frequency
CADD: Combined Annotation Dependent Depletion
TSI: Tuscan Italians
qPCR: Quantitative PCR
ROC: Receiver Operating Characteristic
FPD: Familial PD
SPD: Sporadic PD
