# Supplementary material for: Identification of sixteen novel candidate genes for late onset Parkinson’s disease
Source: Mol Neurodegener. 2021 Jun 21;16:35. doi: 10.1186/s13024-021-00455-2 (PMC8215754; doi:10.1186/s13024-021-00455-2)
Supplement: Supplementary file 1 — Additional file 1: Figure 1S. Evolutionary conservation study of the likely pathogenic variants identified in the 16 novel Parkinson’s disease candidate genes. The affected residues are reported in bold underlined. Figure 2S. Single channel images of human and mouse immunostainings shown in Fig. 5. (a-d) Single channel images showing expression of TH in combination with TOMM22, GIPC1, ZSCAN21, SLC25A39 and HSPA8 in human SN neurons (a) and in pretectal SN (b) mesencephalic SN (c) and VTA (d) mouse neurons. Images in (a) correspond to those reported in Fig. 5a; images in (b-d) correspond to those reported in Fig. 5c, e, g, respectively. Figure 3S. Expression analysis of TOMM22, GIPC1, ZSCAN21, SLC25A39 and HSPA8 in rat DA neurons. a-e Immunohistochemistry experiments performed on P60 rat brain sections showed that TOMM22, GIPC1, ZSCAN21, SLC25A39 and HSPA8 were co-expressed with TH in mesencephalic DA neurons of the SN (b, c) and VTA (d, e). Images in (c, e) correspond to magnification of the boxed area reported in (b, d). Sections in (a) are low magnification images showing only TH immunostaining and corresponding to the same sections reported in (b-e). Scale bars correspond to 100 μm. Abbreviations: SN stands for substantia nigra and VTA for ventral tegmental area. In (c, e) are shown merge and single channel images. Table S1. Cohort description. Table S2. List of primers used for gene expression analysis by quantitative PCR (qPCR). Table S3. Rare disruptive variants (MAF ≤ 0.001, CADD phred ≥ 20) identified in the 16 novel PD candidate genes in the validation cohorts. Table S4. Rare variants (MAF ≤ 0.001) in the 26 PD candidate genes in affected and healthy individuals of the Italian cohort. Table S5. GBA variants annotated in Italian cohort of patients and controls. [file 13024_2021_455_MOESM1_ESM.pdf]

Identification of sixteen novel candidate genes for late onset Parkinson's disease

Alessandro Gialluisi PhD[1,#], Mafalda Giovanna Reccia PhD[1,#], Nicola Modugno MD[1], Teresa Nutile PhD[2], Alessia Lombardi PhD[1], Luca Giovanni Di Giovannantonio PhD[2], Sara Pietracupa MD[1], Daniela Ruggiero PhD[1,2], Simona Scala PhD[1], Stefano Gambardella MD[1,3], International Parkinson's Disease Genomics Consortium (IPDGC)[4], Licia Iacoviello MD PhD[1,5], Fernando Gianfrancesco PhD[2], Dario Acampora PhD[2], Maurizio D'Esposito PhD[2], Antonio Simeone PhD[2], Marina Ciullo PhD[1,2] and Teresa Esposito PhD[1,2,*].

Supplementary data

Figures and Tables

AIMP2

```
      R26Q  D86E  P165F/V  P177L  T203M   I213T
Human MYRL…DADLD….ENLLK……PRQD……IQTMC……GNIAR
Chimp MYRL…DADLD……ENLLK……PRQD….VQTMC……GNIAR
Mouse MYRL…DADLD……ENLVK……SRHE….VQTMC……GNIAR
Fish  MYKL…DADLD……APLLS……ARHR….TQNMC……GNVAR
```

CHMP1A

```
                                A66V
           T11M    A31V  R64W  R68H    S127L
Human      KFTAK…EQAKV…WLRMASRVD…HTSVM
Chimp      KFTAK…EQAKV…WLRMASRVD.HTSVM
Mouse      KFTAK…EQAKV…WLRMASRVD…HTSVM
Fish       RFTSK…EQAKV…WLRMASRVD…HTSVM
Insects    RFTAK…QKAKV…YLRMSSKVD…RISVM
```

GIPC1

```
              V59M       T208M    K292fs
          P50R     A152S      P214S     E316K
Human    PPPP. LVF…GAG…FTL.TEP…KDKR…DEF
Chimp    PPPP….LVF GAG…FTL.TEP KDKR…DEF
Mouse    PPPP….LVF.GAG…FTL.TEP KDKR…DEF
Fish     PPPH….LVF.GAG…FFL.VEP KDKK…DEF
Insects……..….LVF.GAG…FTL.VEP………….....
```

HMOX2

```
       D17N  R76W  F205L  R234L
Human  HDR……HRE….IFN………MRK
Chimp  HDR……HRK …IFN. …MRK
Mouse  HDR……HRK   IFS    IRK
Fish   HDR……HRR …VFE…….VRK
```

TOMM22

```
      P13L   D35N
Human GEPQS..EEDDD…
Chimp GEPQS …EEDDD.
Mouse -EPLS….EEDDD
Fish  SQPQS ……………..  …
```

HSPA8

```
            V18L      V83I      I212S
Human       SCVGV……..AVVQS………LTIED
Chimp       SCVGV……..AVVQS………LTIED
Mouse       SCVGV …….AVVQS ……LTIED
Fish        SCVGV …….AVVQS ……LTIED
Insect      SCVGV …….AVVQS ……LTIED
C.elegans   SCVGV …….AVVQS ……LTIED
Bacteria    SCVGV …….AVVQS ……LTIED

            I253M    Q585K     A636T
Human       KDISE…..KNQTA…..GGASS
Chimp       KDISE…..KNQTA ….GGASS
Mouse       KDISE…..KNQTA ….GGASS
Fish        KDISE…..KNQTA…..GGGST
Insect      KDISE ….ANQLA…..GSGGA
C.elegans   KDISE ….SNQTA……………………
Bacteria    KDISE ….KNQTA…..GGASS
```

ZSCAN21

```
         R67W      G412R
   E29del    P387A     E469G
Human VEE…QLRVL…KPY…TGEK..TGEGE…
Chimp VEE…QLRVL…KPY…TGEK …TGEGE
Mouse AEE…QLRVL…KPY…TGEK….TGEGE
Fish  ……..…RLRTL…KPF…TGEK……TGE..
…
```

TMEM175

```
      R35C R183X A270T    P308L
Human QRM…YRRHV….VYAIV…….TGPRF
Chimp QRM…YRRHV …VYAIV. …TGPRF
Mouse HRM…SRRHI….VYAIV    YGPQF
Fish  HRL…YKYQI …VFAIV ……YGPEF
```

HSPA8

```
          V18L      V83I      I212S
Human     SCVGV…….AVVQS………LTIED
Chimp     SCVGV…….AVVQS………LTIED
Mouse     SCVGV …….AVVQS ……LTIED
Fish      SCVGV …….AVVQS ……LTIED
Insect    SCVGV …….AVVQS ……LTIED
C.elegans SCVGV …….AVVQS ……LTIED
Bacteria  SCVGV …….AVVQS ……LTIED

          I253M    Q585K    A636T
Human     KDISE…..KNQTA…..GGASS
Chimp     KDISE…..KNQTA ….GGASS
Mouse     KDISE…..KNQTA ….GGASS
Fish      KDISE…..KNQTA…..GGGST
Insect    KDISE ….ANQLA…..GSGGA
C.elegans KDISE ….SNQTA…………………
Bacteria  KDISE ….KNQTA…..GGASS
```

IMMT

```
         G107S    H358D    R525S   R622H    K688I
      R31C     S192C    A405V    T588I   N652Y   F708S
Human CRY..SGP SSI…YHE…IAH…RRL…PTI…TRG…RNS…FKL…KFV
Chimp CRR……SGP…SSI…YHE…IAH…RRL…PTI…TRG…RNS…FKL…KFV
Mouse CRR….SGP…SSV…YHE…IAH…RRL…PTI…TRG…RNS…FKL…KFV
Fish  CRG……SGP…EPI…YSE…IAH…RRL…PTE…SRG…RNS…FKL…KFI
```

KIF21B

```
              G92R   R168C G188S   A404V
          F48C   A145T R170C    R211C   S529T
Human   …AFT…AGK…SAQ…RHRRS…TGV…SRTT…KAG…GSP
Chimp   …AFT…AGK…SAQ…RHRRS…TGV…SRTT…KAG…GSP
Mouse   …AFT…AGK…SAQ…RHRRS…TGV…SRTT…KAG…GSP
Fish    …AFT…SGK…SAQ…RHKKS…VGV…SRTT…KTG…………
Insects…AFT…SGK…AAQ…SKCKS…TGV…SRTT…KQG…………

           D638N   R772W   A1079T  R1189Q
       T582M   E742Q   R871L  Y1103C  P1216L
Human   ETD…ADL…YER…QRR…VRQ…DAL…GYA…YRD…SPL
Chimp   ETD.ADL…YER QRR…VRQ…DAL…GYA…YRD…SPL
Mouse   ETD.ADL…YER…QRR…VRQ…DAL…GYA…YRD…SPL
Fish    ETD.ADL…YER…QR…………….DAL…GYA…ECR…SPL
InsectsETE.NDI….EN.QRH……………………………………………….

          R1125Q  D1318E  V1373M
      Y1223C   Q1262L  S1329N  G1447S
Human   SYDR…DRG…SQG….TDE…RSC…KVW…VGK
Chimp   SYDR…DRG…SQG……TDE…RSC…KVW…VGK
Mouse   SYDR…DRG…SQG……TDE…RSC…KVW…IGK
Fish    SYDK…DRG…LQ………TDD…RTC…KVW…TGK
```

KIF24

```
          I58V    R225Fs  F276S
      L9I    S102C   A262T   I324T
Human CLC…LIK…DSP…IRV…EAV…YFDE…MIG
Chimp CLC…LIK…DSP…IRV…EAV…YFDE…MIG
Mouse CLC…LIK…DSP…IRV…EAV…YFDE…MIG
Fish  CLC…LIQ…VSL…IRV…EAV…YFDE…MIG

        I340M  Q491R   R653C
      D339H R376M V588F  Q707R
Human KDIF…KRL…DQE…KVK…VRS…VQT…
Chimp KDIF KRL…DQE…KVK…VRS…VQT…
Mouse KDIF…KRL…DQE…KVK…TRS…VQP…
Fish  KDLF.KRL…DQE…KVK…FKT…IQA…

      E1058G P1188T M1305I Y1352C
Human …LLENP…GFPGK…DEMAE……QLYLT
Chimp …LLENP GFPGK…DEMAE …QLYLT
Mouse …LLETP…TFPRK…DEMAE …QLYLT
```

RHOT2

```
        K50T    C79Y   G250D   G438C
    T47I   I72Fs  E140K   C363Y  G599W
Human VTP…EKV…EIH…VCV…PEI…DGF…LCQ…LGR…VGA
Chimp VTP…EKV…EIH…VCV…PEI…DGF LCQ…LGR…VGA
Mouse VTP…EKV…EIS…ICI…PEI…EGF LCQ…LGN…VGT
Fish  VTP…EKV…EIN…VCV…TEI…EGF…LCQ…LGK…-GA
```

SLC25A3

```
        R43P  K149T  T253N  E313K I328V
Human …SQRPS…QLKAF…VLTLP…REASG…RIIKA
Chimp …SQRPS…QLKAF…VLTLP…RAESG…RIIKA
Mouse …SQRPS…QLKAF…TLTLP…RAESG…RIIKA
Fish  …AQR………QLRDF…TITLP….ESG…RLMKV
```

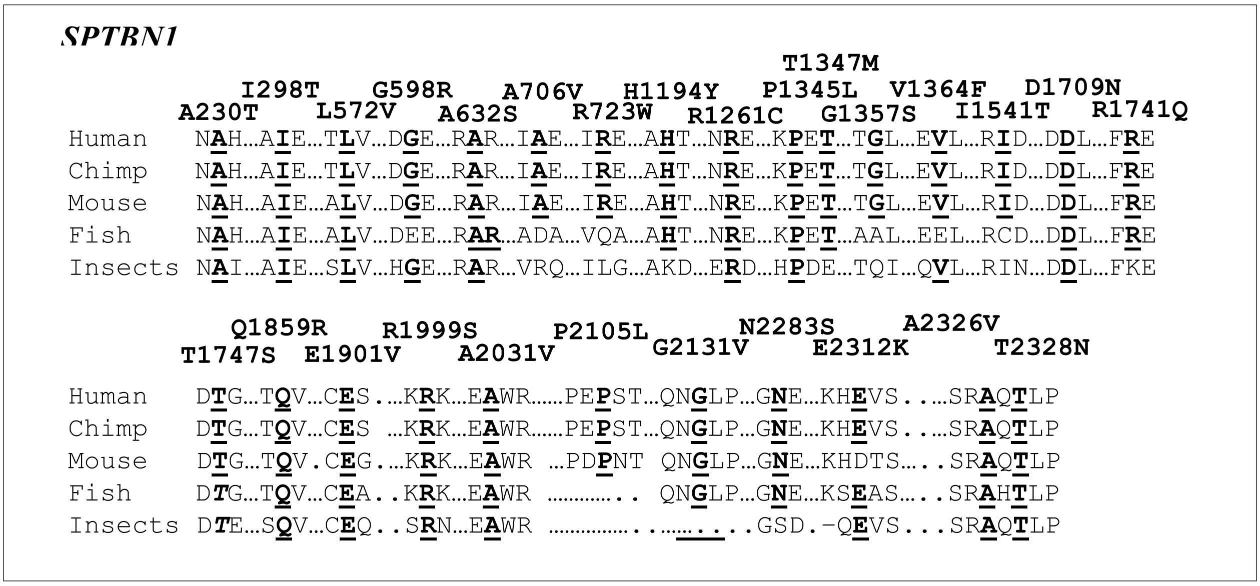

Figure 1S. Evolutionary conservation study of the likely pathogenic variants identified in the 16 novel Parkinson's disease candidate genes. The affected residues are reported in bold underlined.

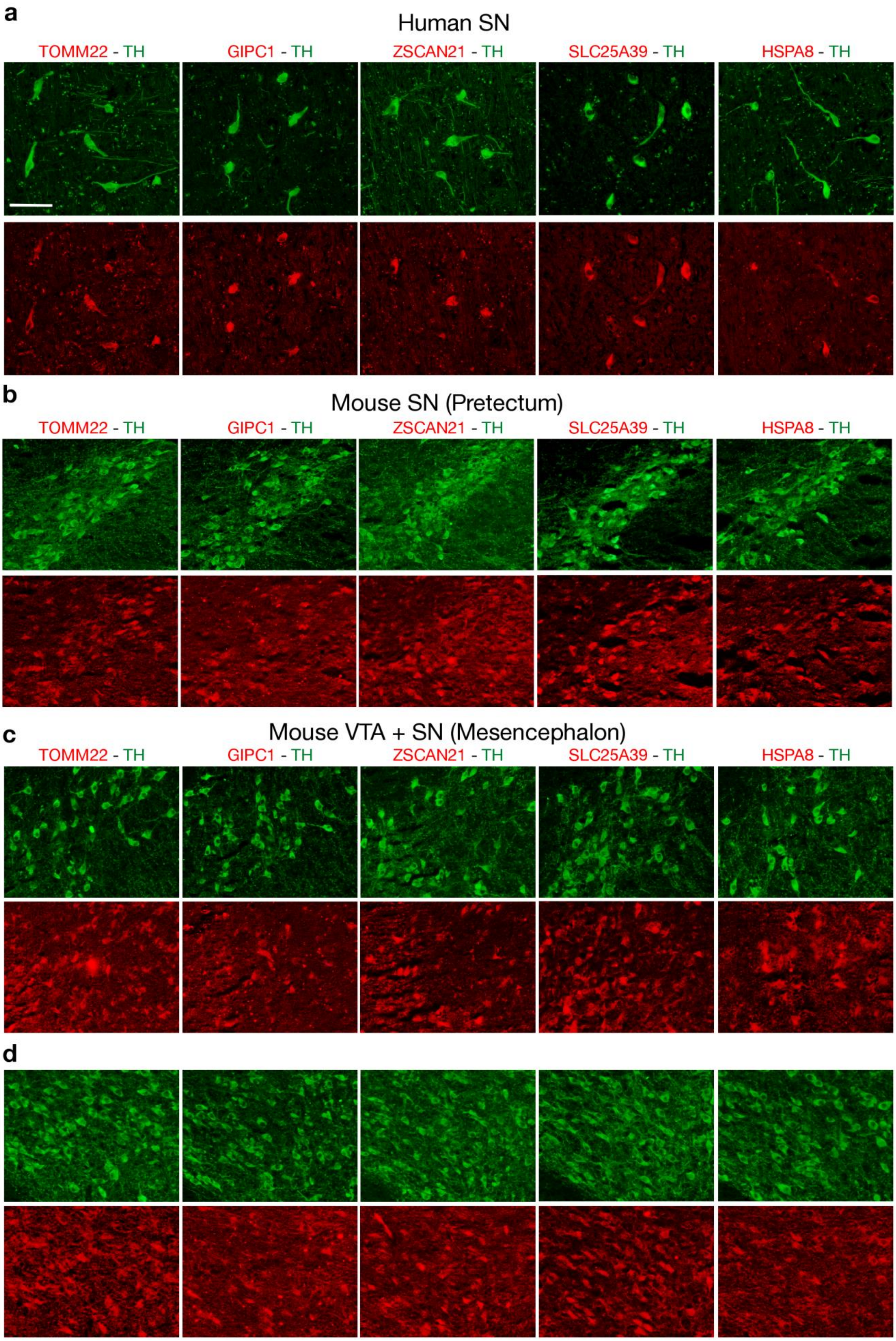
a
Human SN
TOMM22 - TH
GIPC1 - TH
ZSCAN21 - TH
SLC25A39 - TH
HSPA8 - TH
b
Mouse SN (Pretectum)
TOMM22 - TH
GIPC1 - TH
ZSCAN21 - TH
SLC25A39 - TH
HSPA8 - TH
c
Mouse VTA + SN (Mesencephalon)
TOMM22 - TH
GIPC1 - TH
ZSCAN21 - TH
SLC25A39 - TH
HSPA8 - TH
d

Figure 2S. Single channel images of human and mouse immunostainings shown in Fig 5. (**a-d**) Single channel images showing expression of TH in combination with TOMM22, GIPC1, ZSCAN21, SLC25A39 and HSPA8 in human SN neurons **(a)** and in pretectal SN **(b)** mesencephalic SN **(c)** and VTA **(d)** mouse neurons. Images in **(a)** correspond to those reported in Fig. 5a; images in **(b-d)** correspond to those reported in Fig. 5 c, e, g, respectively.

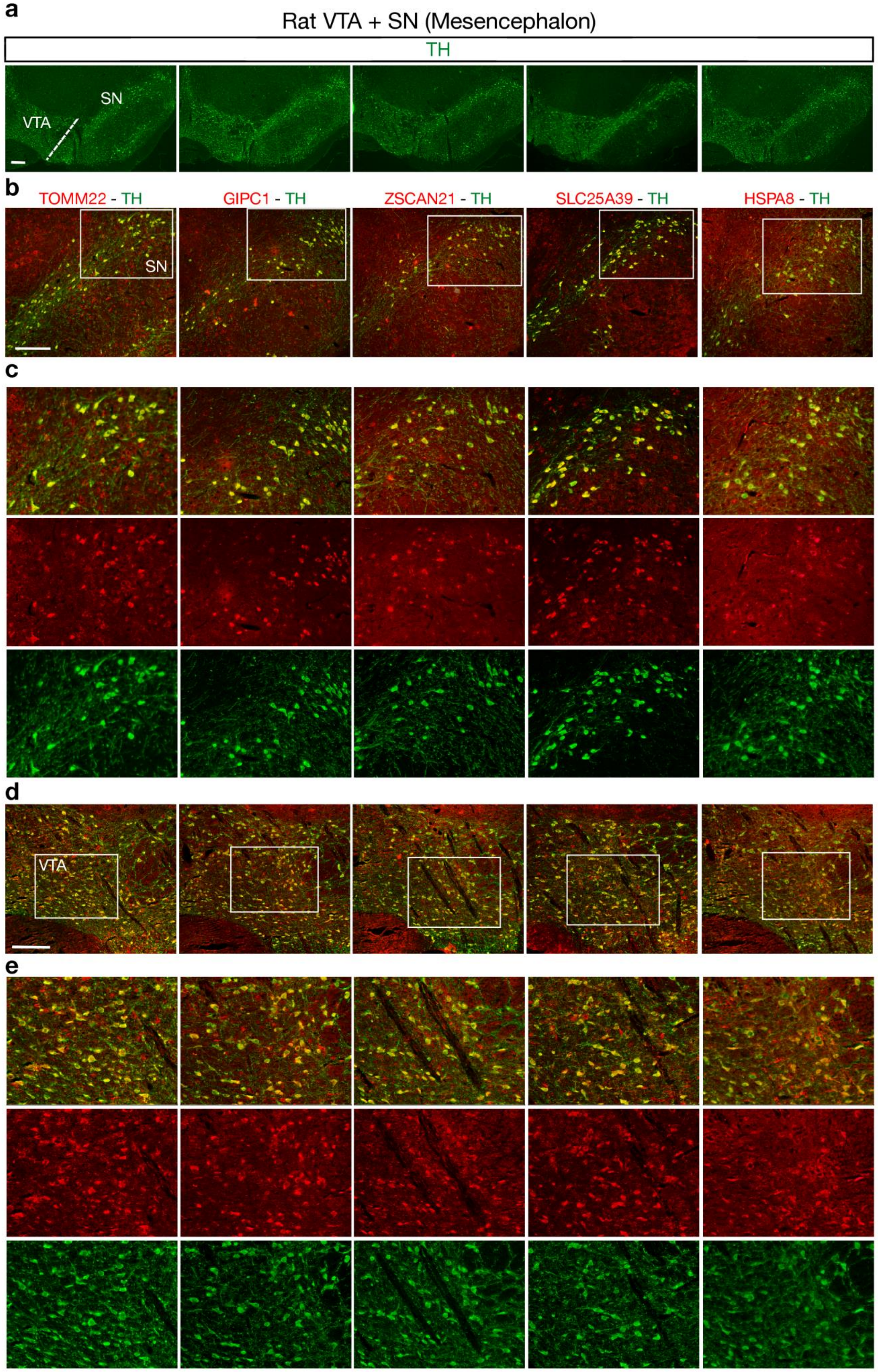
a
Rat VTA + SN (Mesencephalon)
TH
SN
VTA
b
TOMM22 - TH
GIPC1 - TH
ZSCAN21 - TH
SLC25A39 - TH
HSPA8 - TH
SN
c
d
VTA
e

Figure 3S. Expression analysis of TOMM22, GIPC1, ZSCAN21, SLC25A39 and HSPA8 in rat DA neurons. a-e Immunohistochemistry experiments performed on P60 rat brain sections showed that TOMM22, GIPC1, ZSCAN21, SLC25A39 and HSPA8 were co-expressed with TH in mesencephalic DA neurons of the SN (**b, c**) and VTA (**d, e**). Images in (**c, e**) correspond to magnification of the boxed area reported in (**b, d**). Sections in (**a**) are low magnification images showing only TH immunostaining and corresponding to the same sections reported in (**b-e**). Scale bars correspond to 100 µm. Abbreviations: SN stands for substantia nigra and VTA for ventral tegmental area. In (**c, e**) are shown merge and single channel images.

Table S1. Cohort description

Series	n	FPD	SPD	Age mean ± SD	AAO mean ± SD	Type of analysis	Discovery	Validation Analysis	Polygenic load of rare variants	Endophenotype analysis
PD_PIB	47 (23 families)	47	0	66.7 ± 10.39	61.7 ± 8.57	WES	YES	NO	NO	NO
PD_MNI	6 (3 families)	6	0	66.5 ± 3.6	61.3 ± 2.4	WES	YES	NO	NO	YES
PD_MNI	394	172	222	66.57 ± 8.85	58.26 ± 9.74	106 (WES)/288 (NGS_TR)	NO	YES	YES	YES
PD_IPDGC	1148			Nav	40.6	WES	NO	YES	NO	NO
CNT_TSI	107			Nav		WGS	NO	YES	YES	NO
CNT_MNI	38			72.0 ± 3.9		NGS_TR	NO	YES	YES	NO
CNT_MGB	58			77 ± 5.4		NGS_TR	NO	YES	YES	NO
CNT_IPDGC	503			Nav		WES	NO	YES	NO	NO

AAO, Age at onset; FPD, Familial Parkinson's disease; CNT, Control Subjects; MGB, Moli-sani genetic biobank; MNI, Mediterranean Neurological Institute Cohort; PIB, Parkinson Institute Biobank Cohort; SPD, Sporadic Parkinson's disease; SD, Standard Deviation; WES, Whole Exome Sequencing; WGS, Whole genome sequencing; NGS- TR: Next generation sequencing-targeted resequencing.

Table S2. List of primers used for gene expression analysis by quantitative PCR (qPCR)

Gene	Direction	Organism	Sequence 5'-3'	Product PCR size (bp)	Ta (°C)
Aimp2	Fwd	Mouse	GAGCAGGCTAGGAAGCAGTC	136	60°C
	Rev		AACGTGCGATGTTCCCTTCT		
Chmp1a	Fwd	Mouse	AAGGCCGAGAAGGACTCCAA	138	60°C
	Rev		GGCCATTCGGAGCCAATTTAC		
Gipc1	Fwd	Mouse	TCAAGTCGGAGGAGGCTCTG	173	60°C
	Rev		AACCTCGTAATGCCGACAGC		
Hmox2	Fwd	Mouse	AGGGCAGCACAAACTACTCA	169	60°C
	Rev		TCTGCTCGGTCATGTGCTTC		
Hspa8	Fwd	Mouse	CGTACCTCGGAAAGACCGTT	143	60°C
	Rev		TAGCAGCAGCAGTTGGTTCA		
Immt	Fwd	Mouse	TCTGGGTTGACTGCTGGTAAG	164	60°C
	Rev		GCAGAACCAAGAACCATCCC		
Kif21b	Fwd	Mouse	GGGCATCGACGAACGCAA	159	60°C
	Rev		TTGATGTTGGACCTGCGGTG		
Kif24	Fwd	Mouse	ACAGATAGCTGGCCTTCGAG	133	60°C
	Rev		GATAGCATGGGAACGGGAGG		
Man2c1	Fwd	Mouse	TCCCTATGAGAAGGCGGTGC	165	60°C
	Rev		ACAAGGCTTTCTCCATCGCTT		
Pitx3	Fwd	Mouse	TTATCGGACGCAGGCACTCCAC	76	60°C
	Rev		TCTCCGAGTCACTGTGCTCCT		
Rhot2	Fwd	Mouse	GCGCGGAGGAGATAACCATC	159	60°C
	Rev		TGGTCTCCTCAGACACATCG		
Slc25a39	Fwd	Mouse	TCGGCAACCAGCGAATTGA	141	60°C
	Rev		ACCATTTGGGCACAGGTACAG		
Sptbn1	Fwd	Mouse	TTCTGCGCTTCCAGATCCAG	157	60°C
	Rev		ATGCCATCCCTCCAGCTAGT		
Tmem175	Fwd	Mouse	ATAGATGATACACTTGCCTTGCTCA	99	60°C
	Rev		GGGGCACATCAGGGAATGTT		
Tomm22	Fwd	Mouse	TTTGACCTCTCGCTCTTCGT	170	60°C
	Rev		GGCCCTAAAAGTATCTGCCGT		
Tvp23a	Fwd	Mouse	GCTCCTCCTCCTGTCCTTTG	105	60°C
	Rev		TGGCTTTTCCCATCCTCGTC		
Zscan21	Fwd	Mouse	GGATCCAAGAAAGCGCCAAG	127	60°C
	Rev		AGGAATCATGGGGATGACGG		
Gapdh	Fwd	Mouse	TGGAGAAACCTGCCAAGTAT	198	60°C
	Rev		CATACCAGGAAATGAGCTTG		

bp, base pairs; Fwd, Forward; PCR, Polimerase Chain Reaction; Rev, Reverse; Ta, Annealing Temperature

Table S3. Rare disruptive variants (MAF≤0.001, CADD phred≥20) identified in the 16 novel PD candidate genes in the validation cohorts

CHR	Genomic position (hg19)	dbSNP	Gene	RefSeq	Exon/ intron	Nucleotide Change	AA Change	Exonic function	AF_PD Italian	AF_CNT Italian	AF_PD IPDGC	AF_CNT IPDGC	MAF Max in public data sets	CADD phred
7	6049071		AIMP2	NM_006303	exon1	c.G77A	p.R26Q	NSV	0.001	0	0	0	NA	24
7	6054834	rs140850823	AIMP2	NM_006303	exon2	c.C193T	p.R65C	NSV	0.001	0	0	0	0.0006	26
7	6054899		AIMP2	NM_006303	exon2	c.C258G	p.D86E	NSV	0	0	0.00075	0	NA	26
7	6057595		AIMP2	NM_006303	exon3	c.C493G	p.L165V	NSV	0.001	0	0	0	NA	28
7	6057595		AIMP2	NM_006303	exon3	c.C493T	p.L165F	NSV	0.001	0	0	0	NA	32
7	6057632		AIMP2	NM_006303	exon3	c.C530T	p.P177L	NSV	0.001	0	0	0	NA	23
7	6062967	rs766467029	AIMP2	NM_006303	exon4	c.C608T	p.T203M	NSV	0	0	0.00047	0	0.00003	22
16	89715807	rs199628110	CHMP1A	NM_001083314	exon3	c.C184T	p.R62C	NSV	0.001	0	0	0	0.0005	NA
16	89715813	rs201479143	CHMP1A	NM_001083314	exon3	c.G178A	p.V60I	NSV	0.002	0	0	0	0.001	NA
16	89715864	rs200606123	CHMP1A	NM_001083314	exon3	c.C127T	p.R43X	stop	0.001	0	0	0	0.0002	NA
16	89718050	rs367804633	CHMP1A	NM_002768	exon3	c.C32T	p.T11M	NSV	0.001	0	0.0005	0	0.00008	24
16	89715770	rs200470789	CHMP1A	NM_002768	exon4	c.A241T	p.T81S	NSV	0	0	0.00059	0	0.0005	21
16	89715808	rs375600636	CHMP1A	NM_002768	exon4	c.G203A	p.R68H	NSV	0	0	0.00057	0	0.00003	34
16	89715814	rs767659184	CHMP1A	NM_002768	exon4	c.C197T	p.A66V	NSV	0	0	0.00056	0	0.00003	33
16	89715821	rs371497296	CHMP1A	NM_002768	exon4	c.C190T	p.R64W	NSV	0	0	0.0016	0	0.00009	34
16	89713096	rs61730919	CHMP1A	NM_001083314	exon5	c.C388T	p.H130Y	NSV	0.002	0	0	0	0.0007	NA
16	89713612	rs201945919	CHMP1A	NM_002768	exon5	c.C380T	p.S127L	NSV	0.001	0	0.0007	0	0.00007	22
16	89712459		CHMP1A	NM_001083314	exon6	c.T586A	p.C196S	NSV	0.001	0	0	0	NA	NA
19	14593614	rs755126341	GIPC1	NM_005716	exon4	c.G175A	p.V59M	NSV	0	0	0.0012	0	0.00006	29
19	14593640	rs773237853	GIPC1	NM_005716	exon4	c.C149G	p.P50R	NSV	0	0	0.0021	0	0.00004	23
19	14591425	rs1477574103	GIPC1	NM_005716	exon5	c.G454T	p.A152S	NSV	0	0	0.00083	0	0.00005	25
19	14591546	rs550263336	GIPC1	NM_005716	exon5	c.G333C	p.K111N	NSV	0	0	0.00041	0	0.0004	26
19	14591132	rs1311846207	GIPC1	NM_005716	exon6	c.C640T	p.P214S	NSV	0.003	0	0	0	NA	26
19	14591149	rs757818963	GIPC1	NM_005716	exon6	c.C623T	p.T208M	NSV	0	0	0.00085	0	0.00001	28
19	14589284	rs746037324	GIPC1	NM_005716	exon9	c.G946A	p.E316K	NSV	0	0	0.00051	0	0.00001	32
16	4556945	rs200960043	HMOX2	NM_001286271	exon2	c.G49A	p.D17N	NSV	0	0	0.00071	0	0.00004	22
16	4556960	rs11542539	HMOX2	NM_001127205	exon3	c.A151G	p.T51A	NSV	0.001	0	0	0	0.000008	24

16	4557768	rs147603390	HMOX2	NM_001286271	exon3	c.C172T	p.R58C	NSV	0	0	0.00052	0	0.0003	25
16	4559416	rs145119833	HMOX2	NM_001286271	exon4	c.T613C	p.F205L	NSV	0.001	0	0	0	0.00007	27
16	4559504		HMOX2	NM_001286271	exon4	c.G701T	p.R234L	NSV	0	0	0.00055	0	NA	22
11	122931981		HSPA8	NM_006597	exon2	c.G52C	p.V18L	NSV	0.001	0	0	0	NA	24
11	122930542		HSPA8	NM_006597	exon5	c.C759G	p.I253M	NSV	0.001	0	0	0	NA	21
11	122930666		HSPA8	NM_006597	exon5	c.T635G	p.I212S	NSV	0.002	0	0	0	NA	27
11	122929971	rs367639438	HSPA8	NM_006597	intron5	c.1121-2->T		splicing	0.001	0	0	0	0.0005	NA
11	122928477		HSPA8	NM_006597	exon8	c.G1906A	p.A636T	NSV	0.001	0	0	0	NA	26
11	122928962		HSPA8	NM_006597	exon8	c.C1753A	p.Q585K	NSV	0.001	0	0	0	NA	25
2	86385802		IMMT	NM_001100169	exon10	c.C1072G	p.H358D	NSV	0	0	0.00044	0	NA	25
2	86378604	rs765530957	IMMT	NM_001100169	exon12	c.C1214T	p.A405V	NSV	0	0	0.00054	0	0.000008	32
2	86373278	rs560315070	IMMT	NM_001100169	exon14	c.C1573A	p.R525S	NSV	0	0	0.00043	0	0.0002	34
2	86371542	rs200212976	IMMT	NM_001100169	exon15	c.T2123C	p.F708S	NSV	0	0	0.00061	0	0.0002	32
2	86371575	rs201492183	IMMT	NM_001100169	exon15	c.T2090C	p.I697T	NSV	0.003	0	0	0	0.0003	23
2	86371602		IMMT	NM_001100169	exon15	c.A2063T	p.K688I	NSV	0.001	0	0	0	NA	23
2	86371711	rs751220994	IMMT	NM_001100169	exon15	c.A1954T	p.N652Y	NSV	0	0	0.0006	0	0.00003	27
2	86371800	rs376714187	IMMT	NM_001100169	exon15	c.G1865A	p.R622H	NSV	0	0	0.00066	0	0.00002	34
2	86371902	rs372854470	IMMT	NM_001100169	exon15	c.C1763T	p.T588I	NSV	0	0	0.0004	0	0.00001	23
2	86400875	rs376275324	IMMT	NM_001100169	exon4	c.G319A	p.G107S	NSV	0.001	0	0	0	0.0002	22
2	86397949	rs368537238	IMMT	NM_001100169	exon6	c.C575G	p.S192C	NSV	0	0	0.002	0	0.0001	23
1	200969617	rs757244028	KIF21B	NM_001252100	exon11	c.G1586C	p.S529T	NSV	0	0	0.00056	0	0.00005	23
1	200969033	rs553956181	KIF21B	NM_001252100	exon12	c.C1745T	p.T582M	NSV	0.001	0	0	0	0.0002	23
1	200967677	rs759573082	KIF21B	NM_001252100	exon14	c.G1912A	p.D638N	NSV	0.001	0	0	0	0.00001	24
1	200965377	rs750865418	KIF21B	NM_001252100	exon15	c.G2224C	p.E742Q	NSV	0	0	0.00075	0	NA	25
1	200961481	rs1439917989	KIF21B	NM_001252100	exon16	c.C2314T	p.R772W	NSV	0	0	0.00077	0	NA	35
1	200960120		KIF21B	NM_001252100	exon18	c.C2612T	p.R871L	NSV	0	0	0.00053	0	NA	24
1	200978515		KIF21B	NM_001252100	exon2	c.T143G	p.F48C	NSV	0.001	0	0	0	NA	27
1	200957662	rs200143132	KIF21B	NM_001252100	exon23	c.C3309A	p.Y1103X	stop	0	0	0.0092	0	NA	36
1	200957663		KIF21B	NM_001252100	exon23	c.A3308G	p.Y1103C	NSV	0.001	0	0	0	NA	23
1	200956172	rs746216671	KIF21B	NM_001252100	exon25	c.G3566A	p.R1189Q	NSV	0	0	0.00065	0	0.00008	24
1	200955987	rs747677870	KIF21B	NM_001252100	exon26	c.G3674A	p.R1225Q	NSV	0	0	0.00089	0	0.00003	32

1	200955993	rs375526424	KIF21B	NM_001252100	exon26	c.A3668G	p.Y1223C	NSV	0.001	0	0	0	0.0001	26
1	200956014	rs775691918	KIF21B	NM_001252100	exon26	c.C3647T	p.P1216L	NSV	0	0	0.00082	0	0.00001	28
1	200954005		KIF21B	NM_001252100	exon27	c.A3785T	p.Q1262L	NSV	0.001	0	0	0	NA	22
1	200950113	rs1417252859	KIF21B	NM_001252100	exon29	c.T3954G	p.D1318E	NSV	0	0	0.0012	0	0.000004	24
1	200977911	rs368704611	KIF21B	NM_001252100	exon3	c.G433A	p.A145T	NSV	0	0	0.00054	0	0.00002	35
1	200978070	rs747201044	KIF21B	NM_001252100	exon3	c.G274A	p.G92R	NSV	0	0	0.0004	0	0.00001	28
1	200948706	rs745419043	KIF21B	NM_001252100	exon30	c.G4117A	p.V1373M	NSV	0	0	0.00059	0	0.000009	34
1	200948837		KIF21B	NM_001252100	exon30	c.G3986A	p.S1329N	NSV	0	0	0.001	0	NA	31
1	200946008	rs773631554	KIF21B	NM_001252100	exon32	c.G4339A	p.G1447S	NSV	0	0	0.001	0	NA	33
1	200944791	rs146030020	KIF21B	NM_001252100	exon33	c.G4450A	p.E1484K	NSV			0.0012	0	0.0004	23
1	200974708		KIF21B	NM_001252100	exon4	c.G562A	p.G188S	NSV	0	0	0.00044	0	NA	27
1	200974762	rs753749629	KIF21B	NM_001252100	exon4	c.C508T	p.R170C	NSV	0	0	0.00044	0	0.000008	34
1	200974768		KIF21B	NM_001252100	exon4	c.C502T	p.R168C	NSV	0	0	0.00044	0	NA	34
1	200972715	rs746619358	KIF21B	NM_001252100	exon8	c.C1211T	p.A404V	NSV	0	0	0.00055	0	0.00002	25
9	34255745		KIF24	NM_194313	exon11	c.C3857delCCC	p.L1287	NFs_del	0	0	0.00051	0	NA	NA
9	34256043	rs754510690	KIF24	NM_194313	exon11	c.C3562A	p.P1188T	NSV	0.002	0	0	0	0.00001	22
9	34256287	rs767559020	KIF24	NM_194313	exon11	c.3317delC	p.S1106fs	Fs_del	0.001	0	0	0	0.00006	NA
9	34256432		KIF24	NM_194313	exon11	c.A3173G	p.E1058G	NSV	0	0	0.00038	0	NA	26
9	34256934	rs148278926	KIF24	NM_194313	exon11	c.A2671T	p.S891C	NSV	0.001	0	0	0	0.001	23
9	34257485	rs768953468	KIF24	NM_194313	exon11	c.A2120G	p.Q707R	NSV	0	0	0.00039	0	0.000008	22
9	34257622	rs771778476	KIF24	NM_194313	exon11	c.1982dupA	p.K661fs	Fs_ins	0.001	0	0	0	0.000008	NA
9	34257648	rs200178053	KIF24	NM_194313	exon11	c.C1957T	p.R653C	NSV	0	0	0.00037	0	0.0002	23
9	34257843	rs528128260	KIF24	NM_194313	exon11	c.A1762T	p.V588F	NSV	0	0	0.00036	0	0.00003	24
9	34255121	rs753823453	KIF24	NM_194313	exon12	c.G3915A	p.M1305I	NSV	0	0	0.00053	0	NA	23
9	34254430	rs754116500	KIF24	NM_194313	exon13	c.A4055G	p.Y1352C	NSV	0	0	0.00039	0	NA	21
9	34311040	rs368653412	KIF24	NM_194313	exon2	c.C305G	p.S102C	NSV	0	0	0.00038	0	0.00008	23
9	34311173	rs749554247	KIF24	NM_194313	exon2	c.A172G	p.I58V	NSV	0	0	0.0004	0	0.0001	24
9	34311320	rs777170244	KIF24	NM_194313	exon2	c.C25A	p.L9I	NSV	0.001	0	0	0	0.00005	27
9	34306279	rs374365344	KIF24	NM_194313	exon3	c.G784A	p.A262T	NSV	0.004	0	0	0	0.0002	32
9	34306389	rs761925363	KIF24	NM_194313	exon3	c.673delA	p.R225fs	Fs_del	0.001	0	0	0	0.00004	NA
9	34297099	rs746293482	KIF24	NM_194313	exon4	c.T827C	p.F276S	NSV	0.001	0	0	0	0.00008	29

9	34290172		KIF24	NM_194313	exon5	c.G1127T	p.R376M	NSV	0	0	0.00059	0	NA	32
9	34290279	rs771948184	KIF24	NM_194313	exon5	c.C1020G	p.I340M	NSV	0	0	0.00077	0	0.00006	23
9	34290284	rs771948184	KIF24	NM_194313	exon5	c.G1015C	p.D339H	NSV	0	0	0.00079	0	0.00006	28
9	34271806		KIF24	NM_194313	intron8	c.1337+1G>A		splicing	0	0	0.00059	0	NA	26
9	34263142		KIF24	NM_194313	exon9	c.A1472G	p.Q491R	NSV	0	0	0.0005	0	NA	23
15	75660844	rs760253802	MAN2C1	NM_001256494	exon1	c.76_80del	p.F26fs	Fs_del	0.001	0	0	0	0.0001	NA
15	75660852		MAN2C1	NM_001256494	exon1	c.T73C	p.Y25H	NSV	0.001	0	0	0	NA	24
15	75653996	rs141487118	MAN2C1	NM_001256494	exon10	c.C1167G	p.I389M	NSV	0.001	0	0	0	0.001	23
15	75653704		MAN2C1	NM_001256494	exon11	c.G1237A	p.E413K	NSV	0	0	0.00043	0	NA	24
15	75653431	rs561636022	MAN2C1	NM_001256494	exon12	c.G1416A	p.M472I	NSV	0	0	0.00047	0	0.001	29
15	75653507	rs199648911	MAN2C1	NM_001256494	exon12	c.G1340A	p.R447Q	NSV	0.001	0	0	0	0.0002	23
15	75652110	rs149931862	MAN2C1	NM_001256494	exon16	c.G1799A	p.R600H	NSV	0	0	0.00045	0	0.0006	22
15	75652111	rs773250077	MAN2C1	NM_001256494	exon16	c.C1798T	p.R600C	NSV	0	0	0.00045	0	NA	23
15	75651750		MAN2C1	NM_001256494	exon17	c.C1978T	p.Q660X	stop	0	0	0.00049	0	NA	36
15	75651094	rs146675988	MAN2C1	NM_001256494	exon19	c.C2248T	p.P750S	NSV	0.003	0	0	0	0.0003	27
15	75660415	rs1433779625	MAN2C1	NM_001256494	exon2	c.A226C	p.T76P	NSV	0.001	0	0	0	0.000004	22
15	75660417		MAN2C1	NM_001256494	exon2	c.C224G	p.P75R	NSV	0	0	0.00091	0	NA	24
15	75650816	rs765312123	MAN2C1	NM_001256494	exon20	c.G2441A	p.R814H	NSV	0	0	0.00049	0	0.00001	25
15	75650555		MAN2C1	NM_001256494	exon21	c.G2585A	p.W862X	stop	0	0	0.00047	0	NA	48
15	75650631	rs142008270	MAN2C1	NM_001256494	exon21	c.C2509T	p.R837W	NSV	0	0	0.00047	0	0.0007	35
15	75648985	.	MAN2C1	NM_001256494	exon23	c.2744_2749del	p.915_917del	NFs_del	0.001	0	0	0	NA	NA
15	75648688	rs775890577	MAN2C1	NM_001256494	exon24	c.C2891T	p.A964V	NSV	0.001	0	0	0	0.000004	22
15	75658862	rs138736683	MAN2C1	NM_001256494	intron5	c.422+1G>A		splicing	0.001	0	0.00091	0	0.0005	21
15	75656475	rs144333944	MAN2C1	NM_001256494	exon6	c.A655G	p.M219V	NSV	0	0	0.00046	0	0.0003	20
15	75654995	rs778977181	MAN2C1	NM_001256494	exon7	c.T885G	p.F295L	NSV	0	0	0.00047	0	0.00002	25
15	75654725	rs200595616	MAN2C1	NM_001256494	exon8	c.C967T	p.R323C	NSV	0	0	0.00043	0	0.001	28
16	721083	rs199677807	RHOT2	NM_138769	exon11	c.G749A	p.G250D	NSV	0	0	0.00067	0	0.00002	24
16	721118	rs138574714	RHOT2	NM_138769	exon11	c.G784A	p.G262S	NSV	0	0	0.00061	0	0.0003	32
16	721762	rs201106544	RHOT2	NM_138769	exon12	c.T935C	p.V312A	NSV	0	0	0.0010	0	0.0006	22
16	721993		RHOT2	NM_138769	exon13	c.G1088A	p.C363Y	NSV	0.001	0	0	0	NA	26
16	722370		RHOT2	NM_138769	exon15	c.G1312T	p.G438C	NSV	0.001	0	0	0	NA	27

16	718531	rs1315175843	RHOT2	NM_138769	exon3	c.C140T	p.T47I	NSV	0.001	0	0	0	NA	31
16	718540	rs776871300	RHOT2	NM_138769	exon3	c.A149C	p.K50T	NSV	0	0	0.00054	0	0.000009	26
16	718691		RHOT2	NM_138769	exon4	c.214_215insC	p.I72fs	Fs_ins	0.001	0	0	0	NA	NA
16	719566	rs895243353	RHOT2	NM_138769	exon5	c.G236A	p.C79Y	NSV	0	0	0.00054	0	NA	26
16	720337	rs371835149	RHOT2	NM_138769	exon7	c.G418A	p.E140K	NSV	0	0	0.00043	0	0.00007	27
17	42397591	rs766153925	SLC25A39	NM_001143780	exon11	c.G937A	p.E313K	NSV	0	0	0.00056	0	0.00004	20
17	42397386	rs189084066	SLC25A39	NM_001143780	exon12	c.C1063T	p.R355W	NSV	0	0	0.00048	0	0.0004	27
17	42397467	rs1310165078	SLC25A39	NM_001143780	exon12	c.A982G	p.I328V	NSV	0	0	0.0005	0	NA	22
17	42400669	rs200614988	SLC25A39	NM_001143780	exon3	c.G128C	p.R43P	NSV	0.001	0	0	0	0.0001	23
17	42398873	rs755813200	SLC25A39	NM_001143780	exon7	c.A446C	p.K149T	NSV	0	0	0.0011	0	0.00002	21
2	54855303		SPTBN1	NM_003128	exon13	c.C1714G	p.L572V	NSV	0.001	0	0	0	NA	27
2	54855381	rs765805170	SPTBN1	NM_003128	exon13	c.G1792A	p.G598R	NSV	0.001	0	0	0	0.00002	25
2	54856165	rs759145266	SPTBN1	NM_003128	exon14	c.G1894T	p.A632S	NSV	0.001	0	0	0	0.000008	23
2	54856388	rs373251033	SPTBN1	NM_003128	exon14	c.C2117T	p.A706V	NSV	0	0	0.00057	0	0.0001	23
2	54856438	rs373885580	SPTBN1	NM_003128	exon14	c.C2167T	p.R723W	NSV	0	0	0.001	0	0.0002	34
2	54859718		SPTBN1	NM_003128	exon17	c.C3580T	p.H1194Y	NSV	0	0	0.00056	0	NA	32
2	54864863	rs765210317	SPTBN1	NM_003128	exon18	c.C3781T	p.R1261C	NSV	0	0	0.00065	0	0.000008	35
2	54871488		SPTBN1	NM_003128	exon20	c.C4034T	p.P1345L	NSV	0.001	0	0	0	NA	34
2	54871494	rs201961584	SPTBN1	NM_003128	exon20	c.C4040T	p.T1347M	NSV	0	0	0.00045	0	0.00007	23
2	54871523	rs757637838	SPTBN1	NM_003128	exon20	c.G4069A	p.G1357S	NSV	0.001	0	0	0	0.00003	20
2	54871544	rs747730085	SPTBN1	NM_003128	exon20	c.G4090T	p.V1364F	NSV	0.001	0	0	0	0.00001	25
2	54873368	rs748217718	SPTBN1	NM_003128	exon23	c.T4622C	p.I1541T	NSV	0	0	0.0011	0	NA	23
2	54876250	rs757703746	SPTBN1	NM_003128	exon25	c.G5125A	p.D1709N	NSV	0.001	0	0	0	0.00002	35
2	54876771	rs761166602	SPTBN1	NM_003128	exon26	c.G5222A	p.R1741Q	NSV	0	0	0.00075	0	0.000008	29
2	54876789	rs373574785	SPTBN1	NM_003128	exon26	c.C5240G	p.T1747S	NSV	0	0	0.00072	0	0.0001	27
2	54877125		SPTBN1	NM_003128	exon26	c.A5576G	p.Q1859R	NSV	0.001	0	0	0	NA	27
2	54880870		SPTBN1	NM_003128	exon27	c.A5702T	p.E1901V	NSV	0.001	0	0	0	NA	25
2	54882221	rs202119435	SPTBN1	NM_003128	exon28	c.T5837C	p.V1946A	NSV	0.001	0			0.001	22
2	54883086		SPTBN1	NM_003128	exon29	c.G5997C	p.R1999S	NSV	0	0	0.00064	0	NA	25
2	54885032		SPTBN1	NM_003128	exon30	c.C6092T	p.A2031V	NSV	0	0	0.00053	0	NA	34
2	54886361	rs140309233	SPTBN1	NM_003128	exon31	c.C6314T	p.P2105L	NSV	0.001	0	0	0	0.00007	23

2	54887108	rs573886474	SPTBN1	NM_003128	exon32	c.G6392T	p.G2131V	NSV	0	0	0.00046	0	0.0002	24
2	54894755	rs879195142	SPTBN1	NM_003128	exon35	c.A6848G	p.N2283S	NSV	0	0	0.0013	0	NA	23
2	54895545	rs540132022	SPTBN1	NM_003128	exon36	c.G6934A	p.E2312K	NSV	0.001	0	0	0	0.0002	23
2	54895588	rs748736138	SPTBN1	NM_003128	exon36	c.C6977T	p.A2326V	NSV	0	0	0.0005	0	0.00002	34
2	54845255	rs760099132	SPTBN1	NM_003128	exon7	c.G688A	p.A230T	NSV	0.001	0	0	0	0.00005	34
2	54849453	rs753318528	SPTBN1	NM_003128	exon9	c.T893C	p.I298T	NSV	0	0	0.00059	0	0.000008	26
4	949644	rs750645874	TMEM175	NM_032326	exon11	c.G808A	p.A270T	NSV	0.001	0	0	0	0.00003	28
4	951692	rs372100086	TMEM175	NM_032326	exon11	c.C923T	p.P308L	NSV	0.002	0	0	0	0.0002	26
4	951812	rs147975675	TMEM175	NM_032326	exon11	c.C1043T	p.S348L	NSV	0.001	0	0	0	0.001	24
4	951982	rs75307864	TMEM175	NM_032326	exon11	c.C1213G	p.L405V	NSV	0.002	0	0	0	0.001	23
4	952049	rs565504915	TMEM175	NM_032326	exon11	c.1281_1282del	p.P427fs	Fs_del	0.002	0	0	0	0.0002	NA
4	941630	rs542936413	TMEM175	NM_032326	exon2	c.C103T	p.R35C	NSV	0.002	0	0	0	0.0002	35
22	39078086		TOMM22	NM_020243	exon1	c.G103A	p.D35N	NSV	0.001	0	0	0	NA	21
16	10912021		TVP23A	NM_001079512	exon2	c.G28T	p.E10X	stop	0	0	0.0008	0	NA	40
7	99654713	rs770822412	ZSCAN21	NM_145914	exon2	c.C85del-GAG	p.E29	NFs_del	0	0	0.0004	0	0.00002	NA
7	99661957	rs375199673	ZSCAN21	NM_145914	exon4	c.G1139A	p.R380Q	NSV	0	0	0.00093	0	0.0003	31
7	99661977	rs149041845	ZSCAN21	NM_145914	exon4	c.C1159G	p.P387A	NSV	0	0	0.00093	0	0.0001	25
7	99662052	rs767375996	ZSCAN21	NM_145914	exon4	c.G1234A	p.G412R	NSV	0	0	0.001	0	0.000008	31
7	99662224	rs776436945	ZSCAN21	NM_145914	exon4	c.A1406G	p.E469G	NSV	0.001	0	0	0	0.00001	27

CHR: Chromosome; hg19: human genome build to which these variants are annotated; dbSNP: reference number in SNP database; ref seq: reference number of the gene transcript; AA Change: amino acid change; PD: Parkinson's disease; IPDGC: International Parkinson's Disease Genetics Consortium; CNT: healthy subject; CADD phred: Combined Annotation Dependent Depletion; Fs_ins: Frame shift insertion; NFs_del: Non Frame shift deletion; NSV: non-synonymous variant; MAF: Minor Allele Frequency; NA: Not Annotated; MAF max in public datasets: highest allelic frequency annotated in public databases including 1000 Genomes Project (AFR. AMR. EAS. EUR. SAS), ExAC browser (NFE. AFR. SAS. EAS and AMR), ESP6500si-v2 (European American and African American population).

Table S4. Rare variants (MAF≤0.001) in the 26 PD candidate genes in affected and healthy individuals of the Italian cohort.

CHR	Genomic position (hg19)	dbSNP	Gene	RefSeq	Exon/ intron	Nucleotide Change	AA Change	Exonic function	CI	AF_PD Italian Cohort	AF_Italian Controls	MAF Max in public data sets	CADD phred
7	6049010	rs139842556	AIMP2	NM_006303	exon1	c.G16C	p.V6L	NSV		0.003	0.002	0.001	24
7	6049071		AIMP2	NM_006303	exon1	c.G77A	p.R26Q	NSV		0.001	0	NA	24
7	6049101		AIMP2	NM_006303	exon1	c.G107A	p.G36D	NSV		0.001	0	NA	0
7	6049124	rs763387407	AIMP2	NM_006303	exon1	c.G130A	p.V44M	NSV		0	0.002	0.00001	23
7	6054834	rs140850823	AIMP2	NM_006303	exon2	c.C193T	p.R65C	NSV		0.001	0	0.0006	26
7	6057595		AIMP2	NM_006303	exon3	c.C493T	p.L165F	NSV		0.001	0	NA	32
7	6057595		AIMP2	NM_006303	exon3	c.C493G	p.L165V	NSV		0.001	0	NA	28
7	6057632		AIMP2	NM_006303	exon3	c.C530T	p.P177L	NSV		0.001	0	NA	23
7	6063036	rs139398981	AIMP2	NM_006303	exon4	c.A677G	p.N226S	NSV		0.001	0.002	0.001	14
7	6063078	rs140208111	AIMP2	NM_006303	exon4	c.C719T	p.A240V	NSV		0	0.002	0.0002	26
7	6063240		AIMP2	NM_006303	exon4	c.C881T	p.T294I	NSV		0.001	0	NA	17
16	89712459		CHMP1A	NM_001083314	exon6	c.T586A	p.C196S	NSV		0.001	0	NA	NA
16	89713051		CHMP1A	NM_001083314	exon5	c.G433A	p.A145T	NSV		0.001	0	NA	13
16	89713096	rs61730919	CHMP1A	NM_001083314	exon5	c.C388T	p.H130Y	NSV		0.002	0	0.0007	NA
16	89713612	rs201945919	CHMP1A	NM_002768	exon5	c.C380T	p.S127L	NSV		0.001	0	0.00007	22
16	89715771		CHMP1A	NM_001083314	exon3	c.G220C	p.D74H	NSV		0	0.002	NA	NA
16	89715807	rs199628110	CHMP1A	NM_001083314	exon3	c.C184T	p.R62C	NSV		0.001	0	0.0005	NA
16	89715813	rs201479143	CHMP1A	NM_001083314	exon3	c.G178A	p.V60I	NSV		0.002	0	0.001	NA
16	89715836	rs374925673	CHMP1A	NM_001083314	exon3	c.G155A	p.R52K	NSV		0.001	0.002	0.0004	16
16	89715864	rs200606123	CHMP1A	NM_001083314	exon3	c.C127T	p.R43X	stop		0.001	0	0.0002	NA
16	89718050	rs367804633	CHMP1A	NM_002768	exon3	c.C32T	p.T11M	NSV		0.001	0	0.00008	24
3	132153462	rs374320101	DNAJC13	NM_015268	exon2	c.A68G	p.K23R	NSV	N	0.001	0	0.0001	19
3	132169528		DNAJC13	NM_015268	exon6	c.G374A	p.R125K	NSV	N	0.001	0	NA	17
3	132169647	rs753475492	DNAJC13	NM_015268	exon6	c.T493G	p.Y165D	NSV	N	0.001	0	0.000008	21
3	132179200		DNAJC13	NM_015268	exon14	c.T1556C	p.V519A	NSV	N	0.001	0	NA	19
3	132199226		DNAJC13	NM_015268	exon26	c.A2786G	p.D929G	NSV	N	0.001	0	NA	22
3	132221296		DNAJC13	NM_015268	exon40	c.A4700G	p.E1567G	NSV	N	0.001	0	NA	23

3	132230035	rs145670794	DNAJC13	NM_015268	exon44	c.C5240T	p.P1747L	NSV	N	0.001	0	0.0005	23
3	132242468		DNAJC13	NM_015268	exon51	c.C5971A	p.Q1991K	NSV	N	0.001	0	NA	23
3	132244526	rs145794875	DNAJC13	NM_015268	exon52	c.G6133A	p.V2045I	NSV	N	0.001	0	0.0001	16
3	132244532		DNAJC13	NM_015268	exon52	c.C6139A	p.P2047T	NSV	N	0.001	0	NA	22
1	65775558		DNAJC6	NM_001256864	exon1	c.G130A	p.G44R	NSV		0.001	0	NA	23
1	65851423	rs146050826	DNAJC6	NM_001256864	exon7	c.G829A	p.A277T	NSV	N	0	0.002	0.0009	19
1	65852598	rs755390221	DNAJC6	NM_001256864	exon8	c.C1099T	p.R367W	NSV	N	0.001	0	0.000008	35
1	65858440		DNAJC6	NM_001256864	exon12	c.T1795C	p.S599P	NSV	N	0.001	0	NA	1
1	65858501	rs139509177	DNAJC6	NM_001256864	exon12	c.G1856A	p.R619H	NSV	N	0.001	0	0.0002	22
1	65864501	rs145175543	DNAJC6	NM_001256864	exon14	c.A2044G	p.S682G	NSV	N	0.001	0	0.0002	21
3	184033960		EIF4G1	NM_182917	exon3	c.G101C	p.S34T	NSV	N	0.001	0	NA	12
3	184039246		EIF4G1	NM_198241	exon10	c.A874G	p.T292A	NSV	N	0.001	0	NA	0
3	184039249		EIF4G1	NM_198241	exon10	c.A877G	p.I293V	NSV	N	0.001	0	NA	0
3	184039286	rs151151194	EIF4G1	NM_198241	exon10	c.G914A	p.R305H	NSV	N	0.001	0.002	0.0004	7
3	184039336		EIF4G1	NM_198241	exon10	c.G964A	p.E322K	NSV	N	0	0.002	NA	23
3	184039709		EIF4G1	NM_198241	exon10	c.C1337A	p.P446H	NSV	N	0.001	0	NA	18
3	184039760		EIF4G1	NM_198241	exon10	c.A1388T	p.E463V	NSV	N	0.002	0	NA	22
3	184040224		EIF4G1	NM_198241	exon11	c.G1594A	p.D532N	NSV	N	0.001	0	NA	29
3	184040390	rs772950492	EIF4G1	NM_198241	exon12	c.C1667T	p.P556L	NSV	N	0.001	0	0.00003	17
3	184041256	rs111396765	EIF4G1	NM_198241	exon15	c.G2149C	p.A717P	NSV	N	0	0.002	0.001	15
3	184045221	rs201711322	EIF4G1	NM_198241	exon24	c.C3646T	p.R1216C	NSV	N	0.001	0	0.0005	35
3	184045416	rs199929867	EIF4G1	NM_198241	exon25	c.C3704T	p.A1235V	NSV	N	0.001	0	0.0002	24
3	184049090	rs149821418	EIF4G1	NM_198241	exon29	c.G4198A	p.G1400R	NSV	N	0.001	0	0.0003	25
3	184052538	rs149821418	EIF4G1	NM_198241	exon33	c.G4642A	p.A1548T	NSV	N	0.001	0	0.0002	4
22	32875089	rs757307611	FBXO7	NM_012179	exon2	c.A244G	p.I82V	NSV	N	0.001	0	0.00001	1
22	32879950	rs373261881	FBXO7	NM_012179	exon3	c.G484A	p.G162S	NSV	N	0	0.002	0.0001	17
22	32879987	rs376455464	FBXO7	NM_012179	exon3	c.C521T	p.S174L	NSV	N	0.001	0	0.0001	24
22	32880067	rs61730783	FBXO7	NM_012179	exon3	c.G601A	p.V201M	NSV	N	0.001	0	0.0009	27
22	32881061	rs376214763	FBXO7	NM_012179	exon4	c.G652A	p.E218K	NSV	N	0	0.002	0.0002	33
22	32881124	rs200924336	FBXO7	NM_012179	exon4	c.A715G	p.M239V	NSV	N	0.002	0	0.00001	13
22	32894389	rs148272407	FBXO7	NM_012179	exon9	c.C1441T	p.R481C	NSV	N	0.001	0	0.0009	29

22	32894401	rs141286570	FBXO7	NM_012179	exon9	c.G1453A	p.V485I	NSV	N	0.001	0.002	0.0007	0
22	32894443	rs369612645	FBXO7	NM_012179	exon9	c.G1495A	p.G499S	NSV	N	0	0.002	0.0007	21
19	14591132	rs1311846207	GIPC1	NM_005716	exon6	c.C640T	p.P214S	NSV		0.003	0	NA	26
19	14593670	rs199741925	GIPC1	NM_005716	exon4	c.C119T	p.S40L	NSV		0.003	0	0.0003	8
16	4546163		HMOX2	NM_001127205	exon2	c.T17A	p.V6D	NSV		0.001		NA	14
16	4546259		HMOX2	NM_001127205	exon2	c.G113T	p.G38V	NSV		0	0.002	NA	1
16	4555533	rs148822872	HMOX2	NM_001127205	exon2	c.C8T	p.A3V	NSV		0.001	0	0.00008	16
16	4556960	rs11542539	HMOX2	NM_001127205	exon3	c.A151G	p.T51A	NSV		0.001		0.000008	24
16	4557846	rs150288371	HMOX2	NM_001286271	exon3	c.A250T	p.M84L	NSV		0.003	0.004	0.001	19
16	4558093	rs765128900	HMOX2	NM_001286271	exon3	c.C497T	p.A166V	NSV		0.001	0.002	0.00004	26
16	4559416	rs145119833	HMOX2	NM_001286271	exon4	c.T613C	p.F205L	NSV		0.001	0	0.00007	27
16	4559434	rs149714752	HMOX2	NM_001286271	exon4	c.G631C	p.A211P	NSV		0.003	0.01	0.0009	21
16	4559710	rs375786109	HMOX2	NM_001286271	exon5	c.G817A	p.A273T	NSV		0.001	0	0.00007	12
11	122928477		HSPA8	NM_006597	exon8	c.G1906A	p.A636T	NSV		0.001	0	NA	26
11	122928962		HSPA8	NM_006597	exon8	c.C1753A	p.Q585K	NSV		0.001	0	NA	25
11	122929971	rs367639438	HSPA8	NM_006597	Intron5	c.1121-2->T		splicing		0.001	0	0.0005	NA
11	122930542		HSPA8	NM_006597	exon5	c.C759G	p.I253M	NSV		0.001	0	NA	21
11	122930666		HSPA8	NM_006597	exon5	c.T635G	p.I212S	NSV		0.002	0	NA	27
11	122930669		HSPA8	NM_006597	exon5	c.C632A	p.T211N	NSV		0.004	0.002	NA	25
11	122931981		HSPA8	NM_006597	exon2	c.G52C	p.V18L	NSV		0.001	0	NA	24
2	74757491		HTRA2	NM_013247	exon1	c.T358G	p.L120V	NSV	N	0.001	0	NA	0
2	74757503		HTRA2	NM_013247	exon1	c.G370A	p.G124R	NSV	N	0.001	0	NA	10
2	74757536		HTRA2	NM_013247	exon1	c.G403C	p.V135L	NSV	N	0.001	0	NA	NA
2	74757598		HTRA2	NM_013247	exon1	c.G465C	p.E155D	NSV	N	0.001	0	NA	23
2	74758769		HTRA2	NM_013247	exon5	c.A985C	p.I329L	NSV	N	0.001	0	NA	23
2	74759841	rs767006508	HTRA2	NM_013247	exon7	c.G1211A	p.R404Q	NSV		0.001	0	0.00001	23
2	86371479	rs200512625	IMMT	NM_001100169	exon15	c.T2186C	p.M729T	NSV		0	0.002	0.001	24
2	86371575	rs201492183	IMMT	NM_001100169	exon15	c.T2090C	p.I697T	NSV		0.003	0	0.0003	23
2	86371602		IMMT	NM_001100169	exon15	c.A2063T	p.K688I	NSV		0.001	0	NA	23
2	86371674	rs534349351	IMMT	NM_001100169	exon15	c.C1991T	p.S664F	NSV		0	0.002	0.001	28
2	86371753	rs745847328	IMMT	NM_001100169	exon15	c.C1912G	p.Q638E	NSV		0.001	0	0.00007	14

2	86371903	rs779931281	IMMT	NM_001100169	exon15	c.A1762G	p.T588A	NSV		0.001	0	0.00001	17
2	86371921		IMMT	NM_001100169	exon15	c.T1744G	p.S582A	NSV		0.001	0	NA	8
2	86374842	rs200238227	IMMT	NM_001100169	exon13	c.A1513G	p.K505E	NSV		0.002	0.005	0.001	23
2	86393750		IMMT	NM_001100169	exon17	c.G670A	p.E224K	NSV		0.006	0.002	NA	27
2	86400875	rs376275324	IMMT	NM_001100169	exon4	c.G319A	p.G107S	NSV		0.001	0	0.0002	22
2	86422481	rs576909274	IMMT	NM_001100169	exon1	c.G25C	p.G9R	NSV		0	0.002	0.001	24
1	200954005		KIF21B	NM_001252100	exon27	c.A3785T	p.Q1262L	NSV		0.001	0	NA	22
1	200955993	rs375526424	KIF21B	NM_001252100	exon26	c.A3668G	p.Y1223C	NSV		0.001	0	0.0001	26
1	200957663		KIF21B	NM_001252100	exon23	c.A3308G	p.Y1103C	NSV		0.001	0	NA	23
1	200960058	rs760098433	KIF21B	NM_001252100	exon18	c.A2674T	p.T892S	NSV		0.001	0	0.00001	0
1	200967533		KIF21B	NM_001252100	exon14	c.G2056A	p.D686N	NSV		0	0.002	NA	33
1	200967638	rs186282104	KIF21B	NM_001252100	exon14	c.G1951A	p.D651N	NSV		0	0.005	0.001	28
1	200967677	rs759573082	KIF21B	NM_001252100	exon14	c.G1912A	p.D638N	NSV		0.001	0	0.00001	24
1	200969033	rs553956181	KIF21B	NM_001252100	exon12	c.C1745T	p.T582M	NSV		0.001	0	0.0002	23
1	200971387		KIF21B	NM_001252100	exon9	c.G1304A	p.R435Q	NSV		0	0.002	NA	31
1	200978515		KIF21B	NM_001252100	exon2	c.T143G	p.F48C	NSV		0.001	0	NA	27
1	200992501		KIF21B	NM_001252100	exon1	c.A11G	p.Q4R	NSV		0.001	0	NA	16
9	34256043	rs754510690	KIF24	NM_194313	exon11	c.C3562A	p.P1188T	NSV		0.002	0	0.00001	22
9	34256287	rs767559020	KIF24	NM_194313	exon11	c.3317delC	p.S1106fs	Fs_del		0.001	0	0.00006	NA
9	34256490		KIF24	NM_194313	exon11	c.G3115A	p.G1039S	NSV		0.001	0	NA	6
9	34256934	rs148278926	KIF24	NM_194313	exon11	c.A2671T	p.S891C	NSV		0.001	0	0.001	23
9	34257622	rs771778476	KIF24	NM_194313	exon11	c.1982dupA	p.K661fs	Fs_ins		0.001	0	0.000008	NA
9	34290241	rs148330049	KIF24	NM_194313	exon5	c.T1058G	p.L353R	NSV		0	0.002	0.0002	23
9	34290263	rs547659170	KIF24	NM_194313	exon5	c.G1036A	p.V346M	NSV		0	0.002	0.001	17
9	34297099	rs746293482	KIF24	NM_194313	exon4	c.T827C	p.F276S	NSV		0.001	0	0.00008	29
9	34306279	rs374365344	KIF24	NM_194313	exon3	c.G784A	p.A262T	NSV		0.004	0	0.0002	32
9	34306389	rs761925363	KIF24	NM_194313	exon3	c.673delA	p.R225fs	Fs_del		0.001	0	0.00004	NA
9	34310875		KIF24	NM_194313	exon2	c.C470T	p.T157I	NSV		0	0.002	0.00008	0
9	34311320	rs777170244	KIF24	NM_194313	exon2	c.C25A	p.L9I	NSV		0.001	0	0.00005	27
12	40631780	rs199567598	LRRK2	NM_198578	exon5	c.C446G	p.T149S	NSV	N	0.002	0	0.00002	13
12	40634334	rs761646404	LRRK2	NM_198578	exon6	c.A621G	p.I207M	NSV	N	0.002	0	0.00001	15

12	40643637		LRRK2	NM_198578	exon8	c.T848C	p.F283S	NSV	N	0.001	0	NA	24
12	40643726		LRRK2	NM_198578	exon8	c.C937T	p.L313F	NSV	N	0.001	0	NA	26
12	40645141		LRRK2	NM_198578	exon9	c.G1066A	p.A356T	NSV	N	0.001	0	NA	27
12	40651124		LRRK2	NM_198578	exon12	c.T1363C	p.S455P	NSV	N	0.002	0	NA	18
12	40657599		LRRK2	NM_198578	exon14	c.G1552A	p.E518K	NSV	N	0.001	0	NA	22
12	40681254	rs199815424	LRRK2	NM_198578	exon20	c.G2602T	p.V868L	NSV	N	0.001	0	0.00002	0
12	40687409		LRRK2	NM_198578	exon21	c.C2752T	p.R918X	stop	P?	0.001	0	NA	36
12	40692110		LRRK2	NM_198578	exon24	c.G3162T	p.L1054F	NSV		0.001	0	NA	23
12	40693014	rs74985840	LRRK2	NM_198578	exon25	c.G3451A	p.A1151T	NSV	NP	0.001	0	0.0003	21
12	40702310	rs772964685	LRRK2	NM_198578	exon29	c.G4001A	p.R1334Q	NSV	N	0.001	0	0.00003	34
12	40702474	rs140743795	LRRK2	NM_198578	exon29	c.G4165A	p.V1389I	NSV	N	0.001	0	0.0003	0
12	40704236	rs33939927	LRRK2	NM_198578	exon31	c.C4321T	p.R1441C	NSV	P	0.003	0	0.00001	26
12	40716141		LRRK2	NM_198578	exon37	c.G5338T	p.V1780F	NSV	N	0.001	0	NA	30
12	40734202	rs34637584	LRRK2	NM_198578	exon41	c.G6055A	p.G2019S	NSV	P	0.01	0	0.0006	35
12	40740665	rs200498558	LRRK2	NM_198578	exon42	c.G6220A	p.V2074I	NSV	N	0.001	0	0.000008	10
12	40745458		LRRK2	NM_198578	exon44	c.A6499T	p.I2167F	NSV	N	0.001	0	NA	21
12	40753147	rs200002022	LRRK2	NM_198578	exon47	c.C6929T	p.T2310M	NSV	N	0.001	0	0.00009	13
12	40757242	rs113511708	LRRK2	NM_198578	exon48	c.C7067T	p.T2356I	NSV	UP	0.002	0	0.0003	16
12	40758777	rs72547983	LRRK2	NM_198578	exon49	c.C7315A	p.L2439I	NSV	UP	0.001	0	0.0001	22
12	40760814	rs281865057	LRRK2	NM_198578	exon50	c.T7397A	p.L2466H	NSV	UP	0.001	0	0.000004	25
12	40760847	rs146428335	LRRK2	NM_198578	exon50	c.G7430A	p.R2477Q	NSV	N	0.001	0	0.0002	10
12	40761466	rs150062967	LRRK2	NM_198578	exon51	c.G7483A	p.V2495I	NSV	N	0.001	0	0.001	17
15	75648688	rs775890577	MAN2C1	NM_001256494	exon24	c.C2891T	p.A964V	NSV		0.001	0	0.000004	22
15	75648985		MAN2C1	NM_001256494	exon23	c.2744_2749del	p.915_917del	NFs_del		0.001	0	NA	NA
15	75649009	rs147299303	MAN2C1	NM_001256494	exon23	c.C2726T	p.A909V	NSV		0	0.002	0.001	25
15	75649207	rs369832470	MAN2C1	NM_001256495	exon22	c.G2584A	p.G862S	NSV		0.001	0.002	0.0001	25
15	75651094	rs146675988	MAN2C1	NM_001256494	exon19	c.C2248T	p.P750S	NSV		0.003	0	0.0003	27
15	75652525	rs377316574	MAN2C1	NM_001256494	exon14	c.C1612T	p.R538W	NSV		0	0.002	0.0001	26
15	75653507	rs199648911	MAN2C1	NM_001256494	exon12	c.G1340A	p.R447Q	NSV		0.001	0	0.0002	23
15	75653996	rs141487118	MAN2C1	NM_001256494	exon10	c.C1167G	p.I389M	NSV		0.001	0	0.001	23
15	75654270	rs148359347	MAN2C1	NM_001256494	exon9	c.G1027A	p.G343R	NSV		0.001	0.002	0.001	32

15	75656523	rs190692217	MAN2C1	NM_001256494	exon6	c.G607A	p.G203R	NSV		0	0.002	0.001	27
15	75656905	rs753036643	MAN2C1	NM_001256494	exon5	c.G524A	p.R175Q	NSV		0.001	0	0.0002	21
15	75658862	rs138736683	MAN2C1	NM_001256494	Intron5	c.422+1G>A		splicing		0.001	0	0.0005	21
15	75658866	rs201676861	MAN2C1	NM_001256494	exon4	c.G419A	p.R140Q	NSV		0	0.002	0.001	14
15	75660415	rs1433779625	MAN2C1	NM_001256494	exon2	c.A226C	p.T76P	NSV		0.001	0	0.000004	22
15	75660423		MAN2C1	NM_001256494	exon2	c.T218G	p.F73C	NSV		0	0.002	NA	24
15	75660482	rs148359347	MAN2C1	NM_001256494	exon2	c.A159C	p.R53S	NSV		0.001	0	0.00001	4
15	75660490		MAN2C1	NM_001256494	exon2	c.C151G	p.P51A	NSV		0.001	0	NA	2
15	75660844	rs760253802	MAN2C1	NM_001256494	exon1	c.76_80del	p.F26fs	Fs_del		0.001	0	0.0001	NA
15	75660852		MAN2C1	NM_001256494	exon1	c.T73C	p.Y25H	NSV		0.001	0	NA	24
6	161771157	rs182893847	PARK2	NM_004562	exon12	c.A1372C	p.M458L	NSV	P	0.001	0	0.001	25
6	161781161	rs778125254	PARK2	NM_004562	exon11	c.C1244A	p.T415N	NSV	UP	0.001	0	0.00001	25
6	161781180		PARK2	NM_004562	exon11	c.G1225T	p.E409X	stop	P	0.001	0	NA	39
6	161969941	rs529808032	PARK2	NM_004562	exon9	c.C1028T	p.P343L	NSV	N	0.001	0	0.00009	24
6	161969984		PARK2	NM_004562	exon9	c.G985T	p.G329C	NSV	N	0.001	0.002	NA	34
6	162206881	rs752922983	PARK2	NM_004562	exon7	c.A794G	p.H265R	NSV	P	0.001	0	0.00004	8
6	162394338		PARK2	NM_004562	exon6	c.G730C	p.V244L	NSV	N	0.002	0	NA	22
6	162394349	rs137853054	PARK2	NM_004562	exon6	c.C719T	p.T240M	NSV	P	0.001	0	0.0003	25
1	8029414	rs538300305	PARK7	NM_007262	exon2	c.G202C	p.D68H	NSV	UP	0.001	0	0.0002	29
1	8029466	rs781094807	PARK7	NM_007262	exon4	c.252+2insA		splicing		0.003	0.002	0.0008	NA
1	8045079	rs71653622	PARK7	NM_007262	exon7	c.G535A	p.A179T	NSV	P	0.002	0.002	0.0009	9
1	20960063		PINK1	NM_032409	exon1	c.G22C	p.G8R	NSV		0	0.002	NA	18
1	20960313		PINK1	NM_032409	exon1	c.G272A	p.G91D	NSV	N	0.001	0	NA	23
1	20960318		PINK1	NM_032409	exon1	c.G277C	p.A93P	NSV		0.002	0	NA	24
1	20964381	rs45604240	PINK1	NM_032409	exon2	c.C434T	p.T145M	NSV	UP	0.001	0	0.0001	13
1	20964505	rs143204084	PINK1	NM_032409	exon2	c.G558C	p.K186N	NSV	P	0.001	0	0.0008	9
1	20964512	rs757581951	PINK1	NM_032409	exon2	c.G565A	p.G189R	NSV	N	0.001	0	0.0001	0
1	20964534	rs138302371	PINK1	NM_032409	exon2	c.C587T	p.P196L	NSV	P	0.002	0	0.0003	17
1	20964573	rs34677717	PINK1	NM_032409	exon2	c.C626T	p.P209L	NSV	P	0.001	0	0.0003	4
1	20964591	rs371854396	PINK1	NM_032409	exon2	c.C644G	p.P215R	NSV	N	0.001	0	0.0001	29
1	20966440		PINK1	NM_032409	exon3	c.C731G	p.A244G	NSV	P	0.001	0	NA	33

1	20971155	rs200949139	PINK1	NM_032409	exon4	c.G949A	p.V317I	NSV	UP	0.001	0	0.001	33
1	20971158	rs139226733	PINK1	NM_032409	exon4	c.A952T	p.M318L	NSV	UP	0.001	0	0.001	24
1	20972108	rs55831733	PINK1	NM_032409	exon5	c.G1015A	p.A339T	NSV	UP	0	0.002	0.001	23
1	20975054	rs757253324	PINK1	NM_032409	exon6	c.A1180G	p.I394V	NSV	N	0.001	0	0.000008	15
1	20975547	rs74315356	PINK1	NM_032409	exon7	c.G1311A	p.W437X	stop	P	0.004	0	0.00001	43
1	20977011	rs531477772	PINK1	NM_032409	exon8	c.G1573A	p.D525N	NSV	P	0.001	0	0.0002	24
16	718509	rs200177302	RHOT2	NM_138769	exon3	c.A118G	p.I40V	NSV		0	0.002	0.001	23
16	718531	rs1315175843	RHOT2	NM_138769	exon3	c.C140T	p.T47I	NSV		0.001	0	NA	31
16	718691		RHOT2	NM_138769	exon4	c.214_215insC	p.I72fs	Fs_ins		0.001	0	NA	NA
16	719558	rs750472415	RHOT2	NM_138769	exon5	c.229_231del	p.77_77del	NFs_del		0	0.002	0.00004	NA
16	720720	rs150725555	RHOT2	NM_138769	exon9	c.T586G	p.S196A	NSV		0	0.002	0.001	18
16	720934	rs145975812	RHOT2	NM_138769	exon10	c.G682A	p.D228N	NSV		0	0.002	0.001	25
16	720944	rs780282687	RHOT2	NM_138769	exon10	c.C692T	p.T231M	NSV		0.001	0	0.00002	14
16	721112	rs200727165	RHOT2	NM_138769	exon11	c.C778T	p.Q260X	stop		0	0.002	0.001	45
16	721182		RHOT2	NM_138769	exon11	c.C848T	p.T283I	NSV		0	0.002	NA	25
16	721993		RHOT2	NM_138769	exon13	c.G1088A	p.C363Y	NSV		0.001	0	NA	26
16	722111	rs150353401	RHOT2	NM_138769	exon14	c.C1125G	p.S375R	NSV		0.001	0.002	0.0008	5
16	722370		RHOT2	NM_138769	exon15	c.G1312T	p.G438C	NSV		0.001	0	NA	27
16	723040	rs547237251	RHOT2	NM_138769	exon18	c.G1640A	p.R547Q	NSV		0.001	0.002	0.0004	21
17	42397458	rs370506274	SLC25A39	NM_001143780	exon12	c.G991C	p.A331P	NSV		0.002	0	0.0002	27
17	42399071	rs146901450	SLC25A39	NM_001143780	exon6	c.C389T	p.T130I	NSV		0.001	0	0.0007	29
17	42399803	rs747485226	SLC25A39	NM_001143780	exon5	c.G308A	p.R103H	NSV		0.001	0	0.00004	12
17	42400666	rs201363204	SLC25A39	NM_001143780	exon3	c.C131T	p.P44L	NSV		0	0.002	0.001	17
17	42400669	rs200614988	SLC25A39	NM_001143780	exon3	c.G128C	p.R43P	NSV		0.001	0	0.0001	23
5	121736821	rs562324777	SNCAIP	NM_001308106	exon2	c.G5A	p.S2N	NSV		0.001	0	0.001	NA
5	121759030	rs773314406	SNCAIP	NM_005460	exon4	c.T598C	p.S200P	NSV	N	0.001	0	0.00001	6
5	121759097	rs147594642	SNCAIP	NM_005460	exon4	c.C665T	p.S222L	NSV		0.001	0	0.001	7
5	121761093	rs868339451	SNCAIP	NM_005460	exon5	c.G1049A	p.G350E	NSV		0.001	0	NA	25
5	121761117	rs371777182	SNCAIP	NM_005460	exon5	c.C1073T	p.A358V	NSV		0.001	0	0.0002	27
5	121786359	rs144492699	SNCAIP	NM_005460	exon10	c.G1817A	p.R606Q	NSV	UP	0.002	0	0.0009	27
5	121786644	rs747135936	SNCAIP	NM_005460	exon10	c.C2102T	p.P701L	NSV		0.001	0	0.000008	26

5	121787074	rs758647740	SNCAIP	NM_005460	exon10	c.2533_2535del	p.845_845del	NFs_del		0.001	0	0.0001	NA
5	121787136	rs150746854	SNCAIP	NM_005460	exon10	c.G2594A	p.R865Q	NSV		0	0.002	0.00007	33
5	121788645	rs764426626	SNCAIP	NM_001242935	exon9	c.T1682G	p.L561R	NSV		0.001	0	0.00006	21
2	54845255	rs760099132	SPTBN1	NM_003128	exon7	c.G688A	p.A230T	NSV		0.001	0	0.00005	34
2	54853194		SPTBN1	NM_003128	exon12	c.G1467T	p.E489D	NSV		0	0.002	NA	25
2	54853349		SPTBN1	NM_003128	exon12	c.T1622C	p.M541T	NSV		0	0.002	NA	19
2	54855303		SPTBN1	NM_003128	exon13	c.C1714G	p.L572V	NSV		0.001	0	NA	27
2	54855381	rs765805170	SPTBN1	NM_003128	exon13	c.G1792A	p.G598R	NSV		0.001	0	0.00002	25
2	54856165	rs759145266	SPTBN1	NM_003128	exon14	c.G1894T	p.A632S	NSV		0.001	0	0.000008	23
2	54858565		SPTBN1	NM_003128	exon16	c.G3381A	p.M1127I	NSV		0	0.002	NA	22
2	54870124	rs766317201	SPTBN1	NM_003128	exon19	c.C3863G	p.S1288C	NSV		0	0.002	0.00003	24
2	54871488		SPTBN1	NM_003128	exon20	c.C4034T	p.P1345L	NSV		0.001	0	NA	34
2	54871523	rs757637838	SPTBN1	NM_003128	exon20	c.G4069A	p.G1357S	NSV		0.001	0	0.00003	20
2	54871544	rs747730085	SPTBN1	NM_003128	exon20	c.G4090T	p.V1364F	NSV		0.001	0	0.00001	25
2	54873397		SPTBN1	NM_003128	exon23	c.A4651G	p.I1551V	NSV		0.001	0	NA	16
2	54873524	rs375275938	SPTBN1	NM_003128	exon23	c.G4778A	p.R1593K	NSV		0.001	0	0.0003	5
2	54876250	rs757703746	SPTBN1	NM_003128	exon25	c.G5125A	p.D1709N	NSV		0.001	0	0.00002	35
2	54876829		SPTBN1	NM_003128	exon26	c.C5280A	p.H1760Q	NSV		0	0.002	NA	14
2	54877125		SPTBN1	NM_003128	exon26	c.A5576G	p.Q1859R	NSV		0.001	0	NA	27
2	54880870		SPTBN1	NM_003128	exon27	c.A5702T	p.E1901V	NSV		0.001	0	NA	25
2	54882221	rs202119435	SPTBN1	NM_003128	exon28	c.T5837C	p.V1946A	NSV		0.001	0	0.001	22
2	54882311	rs140417011	SPTBN1	NM_003128	exon28	c.C5927A	p.S1976Y	NSV		0	0.002	0.001	28
2	54886361	rs140309233	SPTBN1	NM_003128	exon31	c.C6314T	p.P2105L	NSV		0.001	0	0.00007	23
2	54891725	rs200361368	SPTBN1	NM_003128	exon33	c.G6556A	p.A2186T	NSV		0	0.002	0.0005	22
2	54895545	rs540132022	SPTBN1	NM_003128	exon36	c.G6934A	p.E2312K	NSV		0.001	0	0.0002	23
4	941590		TMEM175	NM_032326	exon2	c.G63T	p.R21S	NSV		0	0.002	NA	0
4	941630	rs542936413	TMEM175	NM_032326	exon2	c.C103T	p.R35C	NSV		0.002	0	0.0002	35
4	945017	rs200834686	TMEM175	NM_032326	exon5	c.A313G	p.T105A	NSV		0.002	0	0.001	18
4	946221	rs149575563	TMEM175	NM_032326	exon7	c.G445A	A149T	NSV		0	0.002	0.001	NA
4	947005	rs766495821	TMEM175	NM_032326	exon8	c.T490G	p.F164V	NSV		0	0.002	0.00007	9
4	949644	rs750645874	TMEM175	NM_032326	exon11	c.G808A	p.A270T	NSV		0.001	0	0.00003	28

4	949674		TMEM175	NM_032326	exon10	c.A838G	p.I280V	NSV		0	0.002	NA	24
4	951692	rs372100086	TMEM175	NM_032326	exon11	c.C923T	p.P308L	NSV		0.002	0	0.0002	26
4	951812	rs147975675	TMEM175	NM_032326	exon11	c.C1043T	p.S348L	NSV		0.001	0	0.001	24
4	951878	rs149156711	TMEM175	NM_032326	exon11	c.G1109A	p.R370H	NSV		0.001	0	0.001	0
4	951922		TMEM175	NM_032326	exon11	c.C1153A	p.L385M	NSV		0.001	0	NA	16
4	951982	rs75307864	TMEM175	NM_032326	exon11	c.C1213G	p.L405V	NSV		0.002	0	0.001	23
4	952000		TMEM175	NM_032326	exon11	c.T1231C	p.F411L	NSV		0.001	0	NA	11
4	952049	rs565504915	TMEM175	NM_032326	exon11	c.1281_1282del	p.P427fs	Fs_del		0.002	0	0.0002	NA
4	952238	rs577438263	TMEM175	NM_032326	exon11	c.C1469T	p.T490M	NSV		0	0.005	0.0002	11
22	39078086		TOMM22	NM_020243	exon1	c.G103A	p.D35N	NSV		0.001	0	NA	21
16	10864155	rs371043030	TVP23A	NM_001079512	exon7	c.G616A	p.E206K	NSV		0.002	0	0.0002	7
16	10867236	rs369235815	TVP23A	NM_001079512	exon5	c.A387G	p.I129M	NSV		0.003	0	0.0002	23
16	10868904	rs62000983	TVP23A	NM_001079512	exon3	c.G139A	p.V47I	NSV		0.001	0	0.001	5
7	99654714	rs372435376	ZSCAN21	NM_145914	exon2	c.G85A	p.E29K	NSV		0	0.002	0.0001	11
7	99654719	rs767526646	ZSCAN21	NM_145914	exon2	c.G90C	p.E30D	NSV		0.001	0	0.00003	14
7	99655021	rs747285309	ZSCAN21	NM_145914	exon2	c.G392C	p.G131A	NSV		0.002	0	0.00003	0
7	99655366	rs778353607	ZSCAN21	NM_145914	exon3	c.T445C	p.S149P	NSV		0.001	0	0.00004	0
7	99661443	rs201161088	ZSCAN21	NM_145914	exon4	c.G625A	p.A209T	NSV		0.002	0	0.00003	0
7	99661509		ZSCAN21	NM_145914	exon4	c.A691G	p.N231D	NSV		0.001	0	NA	0
7	99661519		ZSCAN21	NM_145914	exon4	c.A701T	p.E234V	NSV		0.001	0	NA	11
7	99661521		ZSCAN21	NM_145914	exon4	c.G703A	p.A235T	NSV		0.002	0	NA	2
7	99661614	rs146827623	ZSCAN21	NM_145914	exon4	c.G796A	p.V266I	NSV		0.001	0	0.0003	7
7	99662224	rs776436945	ZSCAN21	NM_145914	exon4	c.A1406G	p.E469G	NSV		0.001	0	0.00001	27

CHR: Chromosome; hg19: human genome build to which these variants are annotated; dbSNP: reference number in SNP database; ref seq: reference number of the gene transcript; AA Change: amino acid change; CI: clinical interpretation; P: Pathogenic; UP: uncertain pathogenicity; N: novel; PD: Parkinson's disease; IPDGC: International Parkinson's Disease Genetics Consortium; CADD phred: Combined Annotation Dependent Depletion; Fs_ins: Frame shift insertion; NFs_del: Non Frame shift deletion; NSV: non-synonymous variant; MAF: Minor Allele Frequency; NA: Not Annotated; MAF max in public datasets: highest allelic frequency annotated in public databases including 1000 Genomes Project (AFR. AMR. EAS. EUR. SAS), ExAC browser (NFE. AFR. SAS. EAS and AMR), ESP6500si-v2 (European American and African American population).

Table S5. GBA variants annotated in Italian cohort of patients and controls

CHR	Genomic position (hg19)	dbSNP	Gene	type of variant	CI	Nucleotide Change	AA Change	previous nomenclature	MAF Max in public data sets	CADD phred	AF_SPD	AF_FPD	AF_CNT
1	155204986	rs80356772	GBA	NSV	P	c.G1505A	p.R502H	p.R463H	0.008	29.3	0	0.0029	0
1	155205008	rs368060	GBA	NSV	P	c.G1483C	p.A495P	p.A456P	0.05	23	0	0	0.0024
1	155205518	rs1064651	GBA	NSV	P	c.G1342C	p.D448H	p.D409H	0.00020	22.7	0.0045	0.0029	0
1	155205581	rs149171124	GBA	NSV	NR	c.G1279A	p.E427K	p.E388K	0.00100	23.2	0.0045	0.0058	0
1	155205634	rs76763715	GBA	NSV	P	c.A1226G	p.N409S	p.N370S	0.003	22.7	0.022	0.017	0
1	155206037	rs75548401	GBA	NSV	P	c.C1223T	p.T408M	p.T369M	0.00980	22.2	0.0067	0.011	0.0024
1	155206052	rs121908307	GBA	NSV	P	c.G1208C	p.S403T	p.S364T	0.01	21.3	0.0022	0	0
1	155206161		GBA	NSV	NR	c.C1099T	p.H367Y	p.H328Y	NA	26.4	0.0022	0	0
1	155206167	rs2230288	GBA	NSV	P	c.G1093A	p.E365K	p.E326K	0.02090	17.33	0.009	0.011	00098
1	155206259		GBA	NSV	NR	c.T1001C	p.V334A	p.V295A	NA	26.8	0.0022	0	0
1	155207249	rs367968666	GBA	NSV	P	c.T882G	p.H294Q	p.H255Q	0.00040	6.879	0.0067	0.0058	0.
1	155207965	rs398123534	GBA	NSV	P	c.G721A	p.G241R	p.G202R	0.00002	24.4	0.0022	0	0
1	155207983	rs1064644	GBA	NSV	P	c.T703C	p.S235P	p.S196P	0.00001	11.51	0.0022	0	0
1	155209680		GBA	NSV	NR	c.A304G	p.T102A	p.T63A	Na	1.3	0	0	0.0024
1	155209730	rs77829017	GBA	NSV	P	c.G254A	p.G85E		0.00001	24.8	0.0022	0	0
1	155209862	rs751095441	GBA	NSV	NR	c.G122T	p.R41L		0.01	12	0	0	0.0024

CHR: Chromosome; hg19: human genome build to which these variants are annotated; dbSNP: reference number in SNP database; ref seq: reference number of the gene transcript; AA Change: amino acid change; CI: clinical interpretation; P: Pathogenic; NR: not reported; AF_SPD: allelic frequency in Italian sporadic PD patients; AF_FPD: allelic frequency in Italian familial PD patients; AF_CNT: allelic frequency in Italian controls; CADD phred: Combined Annotation Dependent Depletion; NSV: non-synonymous variant; MAF: Minor Allele Frequency; NA: Not Annotated; MAF max in public datasets: highest allelic frequency annotated in public databases including 1000 Genomes Project (AFR. AMR. EAS. EUR. SAS), ExAC browser (NFE. AFR. SAS. EAS and AMR), ESP6500si-v2 (European American and African American population).

Table S6A. Analysis of PD endophenotypes in 394 Italian PD patients

Series	Scores	0 (%)	1 (%)	≥2 (%)	p (χ^2)
AAO	**Age <50 years**	37 (17.6)	21 (16.5)	17 (26.9)	0.18 (3.39)
	**Age ≥50 years**	173 (82.4)	106 (83.5)	46 (73.1)	reference
UPDRS	**Score <30**	160 (78.0)	94 (74.0)	49 (79.1)	0.64 (0.90)
	**Score ≥30**	45 (22.0)	33 (26.0)	13 (20.9)	reference
MoCA	**Score ≤18**	31 (15.0)	18 (14.6)	10 (16.4)	0.80 (0.44)
	**Score 19-25**	101 (49.0)	58 (47.1)	32 (52.4)	0.67 (0.79)
	**Score ≥26**	74 (36.0)	47 (38.2)	19 (31.2)	reference
LID	**NO**	131 (62.1)	65 (54.1)	30 (46.8)	reference
	**YES**	**80 (37.9)**	**62 (45.9)**	**34 (53.2)**	**0.038 (6.53)**
NMS	**Score ≤54**	110 (53.2)	63 (50.4)	36 (59.1)	reference
	**Score >54**	97 (46.8)	62 (49.6)	25 (40.9)	0.54 (1.22)

AAO: Age at onset; UPDRS: Unified Parkinson's Disease Rating Scale Part III; MoCA: Montreal Cognitive Assessment; LID: Levo-dopa induced Dyskinesia; NMS: non-motor symptoms; χ^2: chi square; p: p-value calculated with Fisher Exact Probability Test. The polygenic variant load (0, 1, ≥2) was calculated considering the variants of the 26 candidate genes identified in the discovery stage of analysis.

Table S6B. Analysis of PD endophenotypes in 394 Italian PD patients including variants in *GBA* gene

Series	Scores	0 (%)	1 (%)	≥2 (%)	p (χ^2)
AAO	**Age <50 years**	**33 (17.4)**	**19 (14.7)**	**23 (28.0)**	**0.044 (6.23)**
	**Age ≥50 years**	156 (82.6)	110 (85.3)	59 (72.0)	reference
UPDRS	**Score <30**	145 (78.0)	99 (77.3)	59 (73.7)	0.75 (0.58)
	**Score ≥30**	41 (22.0)	29 (22.7)	21 (26.3)	reference
MoCA	**Score ≤18**	30 (16.1)	13 (10.5)	15 (19.0)	0.19 (3.32)
	**Score 19-25**	87 (46.7)	64 (51.6)	40 (50.6)	0.66 (0.84)
	**Score ≥26**	69 (37.1)	47 (37.9)	24 (30.4)	reference
LID	**NO**	116 (61.0)	71 (55.0)	39 (47.0)	reference
	**YES**	74 (39.0)	58 (45.0)	44 (53.0)	0.09 (4.75)
NMS	**Score ≤54**	98 (52.7)	68 (52.7)	46 (56.8)	reference
	**Score >54**	88 (47.3)	61 (47.3)	35 (43.2)	0.80 (0.43)

AAO: Age at onset; UPDRS: Unified Parkinson's Disease Rating Scale Part III; MoCA: Montreal Cognitive Assessment; LID: Levo-dopa induced Dyskinesia; NMS: non-motor symptoms; χ^2: chi square; p: p-value calculated with Fisher Exact Probability Test. The polygenic variant load (0, 1, ≥2) was calculated considering the variants of the 26 genes as well as those in *GBA* gene.
